# Visual Hallucinations in Serotonergic Psychedelics and Lewy Body Diseases

**DOI:** 10.1093/schbul/sbaf068

**Published:** 2025-10-06

**Authors:** Nathan H Heller, Frederick S Barrett, Tobias Buchborn, Daniel Collerton, David Dupuis, Adam L Halberstadt, Renaud Jardri, Tehseen Noorani, Katrin H Preller, John-Paul Taylor, Flavie Waters, Brian S Winston, Pantelis Leptourgos

**Affiliations:** Center for Psychedelic and Consciousness Research, Johns Hopkins University School of Medicine, Baltimore, MD 21224, United States; Department of Psychiatry and Behavioral Sciences, Johns Hopkins University School of Medicine, Baltimore, MD 21205, United States; Center for Psychedelic and Consciousness Research, Johns Hopkins University School of Medicine, Baltimore, MD 21224, United States; Department of Psychiatry and Behavioral Sciences, Johns Hopkins University School of Medicine, Baltimore, MD 21205, United States; Department of Neuroscience, Johns Hopkins University School of Medicine, Baltimore, MD 21205, United States; Department of Psychological and Brain Sciences, Johns Hopkins University, Baltimore, MD 21218, United States; Institute of Psychopharmacology, Central Institute of Mental Health, Medical Faculty Mannheim, University of Heidelberg, 68159 Mannheim, Germany; School of Psychology, Faculty of Medical Sciences, Campus for Ageing and Vitality, Newcastle University, Newcastle upon Tyne, NE4 5PL, United Kingdom; Institut national de la santé et de la recherche médicale (INSERM), Paris, 75013, France; Department of Psychiatry, University of California San Diego, San Diego, CA 92093, United States; Research Service, Veterans Affairs San Diego Healthcare System, San Diego, CA 92161, United States; University of Lille, INSERM U-1172, Lille Neurosciences & Cognition Centre (LilNCog), CURE Platform, Fontan Hospital, CHU Lille, 59000, France; School of Pharmacy, University of Auckland, Auckland, 1142, New Zealand; Department of Adult Psychiatry and Psychotherapy, Psychiatric University Clinic Zurich, Zurich, 8032, Switzerland; University of Zurich, Zurich, 8032, Switzerland; Newcastle University, Translational and Clinical Research Institute Newcastle upon Tyne, Tyne and Wear, NE4 5PL, United Kingdom; School of Psychology, University of Western Australia, Nedlands, WA, 6009, Australia; Early Psychosis Program, Black Swan Health, Osborne Park, WA, 6017, Australia; Center for Psychedelic and Consciousness Research, Johns Hopkins University School of Medicine, Baltimore, MD 21224, United States; Department of Psychological and Brain Sciences, Johns Hopkins University, Baltimore, MD 21218, United States; University of Lille, INSERM U-1172, Lille Neurosciences & Cognition Centre (LilNCog), CURE Platform, Fontan Hospital, CHU Lille, 59000, France

**Keywords:** Serotonin receptors, excitatory/inhibitory balance, sensory deprivation, visual hierarchy, hallucinogenesis, phenomenology

## Abstract

**Background and Hypothesis:**

Visual hallucinations (VH) are a core symptom of both Lewy body diseases (LBDs; eg, Parkinson’s disease and dementia with Lewy bodies) and serotonergic psychedelics (SPs; eg, psilocybin and mescaline). While these conditions differ in etiology, overlapping phenomenology, and neural mechanisms suggest shared pathways. This review explores similarities and differences in VH between LBDs and SPs, focusing on phenomenology, cortical function, and serotonergic modulation.

**Study Design:**

This narrative review synthesizes findings from neurology, cognitive neuroscience, and systems neuroscience to compare VH in LBDs and SPs. The literature includes studies with both human subjects and animal models that examine cortical activity patterns, neuromodulatory mechanisms, and VH phenomenology.

**Study Results:**

Both LBDs and SPs exhibit distinct visual aberrations, ranging from minor metamorphopsias to complex hallucinations. Some features in LBDs resemble those induced by SPs (eg, illusory motion and entity encounters), suggesting shared neural mechanisms. Neuroimaging studies indicate a common pattern of hyperactive associative cortex and hypoactive sensory cortex. At the neuromodulator level, SP-induced VH involves serotonin 2A and 1A receptor (5-HT_2A_R and 5-HT_1A_R) modulation, while in LBDs, 5-HT_2A_ receptor upregulation correlates with increased VH, and its inhibition (eg, with pimavanserin) reduces VH. Two shared cortical signatures are highlighted: reduced visual evoked responses and shifts toward visual excitation.

**Conclusions:**

Examining cortical and neuromodulatory similarities between LBD- and SP-induced VH may elucidate the link between sensory degradation, excitation, and hallucinogenesis. Future research should employ real-time neuroimaging of discrete hallucinatory episodes to identify shared mechanisms and develop targeted interventions for LBD hallucinations.

## Introduction

“His first misperceptions occurred when he was in a night club; the skin of the other dancers, even their faces, seemed to be covered with tattoos. At first, he thought the tattoos were real, but they started to glow and then to pulse and writhe; at that point, he realized they must be hallucinatory. As an artist and a psychologist, he was intrigued by this experience—but frightened, too, that it might be the beginning of uncontrollable hallucinations of all sorts.”

– from Oliver Sacks’s *Hallucinations*.^1^

This quote describes the first hallucination of a patient with Parkinson’s disease (PD), a neurodegenerative disorder characterized by abnormal aggregates of the protein alpha-synuclein, known as Lewy bodies. The location of these aggregates varies and serves as a marker of Lewy body disease (LBD) pathology. In PD, they localize to the brainstem or substantia nigra, while in Parkinson’s disease dementia (PDD) and dementia with Lewy bodies (DLB), they appear in limbic, transitional, or cortical regions.^2,3^ As the quote illustrates, visual hallucinations (VH) also mark LBD pathology.^4–10^ The quote highlights three additional key points motivating this review: (1) simpler hallucinations often precede more complex ones in LBDs,^11–15^ (2) these experiences can distress patients,^16–19^ and (3) they resemble hallucinations induced by serotonergic psychedelics.^20^

This last point suggests that serotonergic psychedelics (SPs) like psilocybin, mescaline, lysergic acid diethylamide (LSD), N,N-dimethyltryptamine (DMT), and 2,5-dimethoxy-4-iodoamphetamine (DOI) could model hallucinogenesis in LBDs.^21,22^ While SPs act on multiple neuromodulators,^23,24^ their hallucinatory effects are primarily linked to serotonin 2A and 1A receptors (5-HT_2A_R and 5-HT_1A_R).^25–29^ The 5-HT_2A_R is also implicated in LBD hallucinations,^30–33^ which contribute significantly to clinical and economic burden in these patients,^10^ and predicts earlier institutionalization, caregiver distress, and reduced quality of life.^18,19^ If validated as a model, SPs could be used to investigate hallucinatory mechanisms, develop biomarkers of hallucination proneness, and test interventions for managing these experiences in LBD patients.

While VH in SPs and LBDs may share some etiological overlap, key differences exist. This review from the International Consortium on Hallucinations Research (ICHR) examines these similarities and differences in phenomenology, cortical activity, and serotonergic modulation. Prior ICHR reviews have compared SP-induced VH with those observed in schizophrenia, which are typically part of a broader, multimodal symptom profile dominated by auditory hallucinations.^34,35^ In contrast, VH in neurodegenerative diseases like LBDs are often unimodal and primarily visual.^36^ For this reason, our comparison focuses on unimodal VH. We identify relevant cortical signatures (see Onofrj et al.^21^ for a review of thalamic contributions) and highlight evidence that 5-HT_2A_R and 5-HT_1A_R exert differential effects across the cortical hierarchy, inducing hyper-frontal and hypo-sensory activation. We also suggest that sensory degradation and cortical excitation may play key roles in SP- and LBD-induced VH.

## Phenomenology

### Classifying Hallucinatory Phenomena

To classify hallucination-like experiences, Blom^37^ proposes the following definitions: (1) *hallucinations*—percepts without external stimuli (eg , a person sitting in an empty chair), (2) *illusions*—misidentifications of external stimuli (eg, a chair misperceived as a crouching person), and (3) *metamorphopsias*—distortions of external stimuli (eg, a stationary chair appears to move). Additionally, LBD research often references *minor hallucinations* that are common during early disease stages.^15^ This class can include metamorphopsias, misidentification illusions, and *pareidolia* (ie, seeing meaningful patterns in ambiguous stimuli, such as a face in the clouds), but also *passage hallucinations* (ie, a fleeting, ill-defined entity perceived in peripheral vision) and *presence hallucinations* (ie, the felt sense that an entity is close by). Distinctions are also made between *simple hallucinations* (eg, flashes of light or geometric shapes) and *complex hallucinations* (eg, a whole object or an entire scene). This section compares these classes of phenomenon in LBDs and SPs, highlighting similarities and differences.

### Metamorphopsias

Recent research highlights diverse metamorphopsias in LBDs,^38–46^ with distortions reported in approximately 75% of PD patients. These include altered color, shape, or size perception (ie, metachromatopsia, dysmorphopsia, and micro/micropsia), seeing multiples of a single object (ie, di/polyopia), or perceiving stationary objects as moving (ie, kinetopsia). Similar types of distortions are reported with serotonergic psychedelics.^20,47–49^ While largely anecdotal, limited psychophysical studies have explored some of these psychedelic phenomena systematically.^50–53^

Because visual distortions depend on sensory stimuli, they resemble classic visual illusions,^54^ which can be studied using psychophysical modulation of stimulus and task parameters.^55^ Investigating their origins psychophysically by isolating sensory and perceptual components may be more feasible than studying higher-level hallucinations. Focusing on visual distortions common to both LBDs and SPs could reveal shared neural mechanisms (see Box 1).

Box 1. **Common Hallucinations and Shared Mechanisms**

Neurodegenerative and pharmacological processes affect the visual system in significantly different ways, yet shared mechanisms may underlie *specific* hallucinatory phenomena common to both LBDs and SPs. Illusory motion (kinetopsia) exemplifies this potential. It is reported in ~25% of PD patients^43^ and is a hallmark of psychedelic experiences.^20,47,49^ Convergent psychophysical evidence suggests the involvement of a specific perceptual process in both groups: impairments in higher-order, global-motion integration but not lower-order, local-motion processing.^56–58^ This dissociation implicates the middle temporal (MT) cortex, a region selective for higher-order motion processing. Reduced MT responsiveness is also linked to hallucination proneness in LBD patients.^59–62^ These findings suggest MT hypoactivation may contribute to illusory motion in both groups. Combined psychophysical and neuroimaging evidence could uncover links between other metamorphopsias in LBDs and SPs.

Other hallucinatory phenomena are less viable candidates for this “common phenomenology” approach. Simple hallucinations, such as vivid geometric patterns, are a canonical SP-induced effect.^20,47,49^ In contrast, they are rare in LBDs.^63^ Moreover, these types of simple hallucinations are commonly the result of eye disease (ie, Charles Bonnet syndrome),^64^ which may be concurrent with LBD diagnoses.^65^ This could confound comparisons of simple hallucinations in LBDs and SPs. An even clearer counter-example to this approach is synesthesia, which is commonly reported following SP administration^66^ and never reported in the LBD literature. Ultimately, identifying shared and distinct hallucinatory mechanisms in LBDs and SPs will depend on the careful selection of which phenomena to study.

### Minor Hallucinations

Minor hallucinations have been reported in 42% of newly diagnosed LBD patients and are the most prevalent hallucinatory symptom of these disorders.^13,15,67–69^ These phenomena include passage hallucinations, misidentification illusions, and pareidolias.^5,12,15,70^

While not emphasized in the psychedelic literature, anecdotal accounts of these phenomenon can be found in online reports of SP-experience: “*I began experiencing pareidolia, seeing faces everywhere, mostly Aztec and Inuit art looking faces, but also some angry looking faces in the bark of trees.”* (pareidolia account from Erowid Experience ID 98005; Published Nov 9, 2022); “*To further complicate matters, plants and rocks constantly morphed into animals and vice versa. I can’t recall exactly how many snakes/tree roots I stepped on or how many squirrels/trees I bumped into.*” (misidentification account from Erowid Experience ID 110580; Published June 3, 2017); “*My eyes were also playing tricks on me. For example, I kept thinking I saw somebody walk by the entrance of my room out of the corner of my eyes, and then I would quickly look and nobody was there.*” (passage hallucination account from Erowid Experience ID 46857; Published Nov 4, 2005). Moreover, a recent semantic analysis of nearly 40,000 psychedelic experience reports found that peripheral hallucinations (i.e., resembling passage hallucinations) were relatively common across compounds,^49^ despite little prior attention.^20,47,48^ This highlights the need for more quantitative methods to uncover underrecognized yet widespread features of psychedelic experience.

Presence hallucinations, another minor hallucination common to LBDs,^12^ occurs in a range of non-clinical contexts,^71,72^ including during bereavement,^73^ religious practice,^74,75^ and SP administration.^76^ Ethnographic research on shamanic uses of ayahuasca found that cultural and contextual expectations can shape the identity of the perceived presence.^77,78^ This effect of expectation parallels results from LBD research, were approximately 50% of patients recognize the identity of a felt presence.^12^

### Complex hallucinations

In LBDs, complex hallucinations occur at rates between 22% and 38%^13^ and often involve individual entities (objects, animals, or people) but can occasionally transform the entire visual scene.^79–81^ To a first approximation, they resemble SP-induced complex hallucinations^47,82-84^ though SP content can be more fantastical,^83^ whereas LBD content tends to be mundane though sometimes distorted.^81^ It is unclear if these differences reflect distinct neural mechanisms or contextual expectations.^77,78^

Even different SP compounds induce different visual effects,^49^ with entities more commonly encountered during DMT experiences compared with others.^24^ Strassman (1995) reported that 50% of participants encountered entities after high-dose DMT, a figure supported by larger studies describing encounters with guides, spirits, or aliens.^85–89^ This propensity of entity-inducing compounds may also result from either distinct neural mechanisms or contextual expectations. Systematic interviews with LBD patients who have experienced SP-induced hallucinations prior to their diagnosis, ideally from a range of SP compounds, could provide valuable insights into these phenomenological similarities and differences (see Suzuki et al.^90^ for a model-based approach).

### A Hypothetical Common Pathway?

In LBDs, the emergence of visual distortions and/or minor hallucinations often, though not always, signals the start of a disease progression toward more complex hallucinations. A similar progression from simple to complex VH can occur with SPs, though over hours rather than years.^48^ Curiously, sleep deprivation follows a comparable trajectory, transitioning from visual distortions to complex phenomena over days.^91^ Future research might explore whether these trajectories reflect superficial similarities or reveal shared mechanisms that naturally progress from lower-order to higher-order regions of the visual hierarchy, despite unfolding over different timescales. In general, careful comparisons between LBD and SP hallucinations, such as those outlined in Table 1, may help identify which perceptual phenomena reflect shared underlying mechanisms and which are likely to diverge across clinical and pharmacological contexts.

**Table 1.** Possibility of Comparing Hallucinatory Phenomenology in LBDs and SPs

Perceptual effect	Lewy body diseases	Serotonergic psychedelics	Comparison
Metamorphopsias	Commonly reported^38–46^ Prevalence (PD): ~75%^44^	Commonly reported^20,47–49,82^ Prevalence: NA	Possible
Synesthesia	Not reported Prevalence: NA	Commonly reported^20,49,66^ Prevalence: ~50%^66^	NA
Simple Hallucinations	Rarely reported^92^ Prevalence: NA	Commonly reported^20,47–49,82^ Prevalence: NA	Difficult
Pareidolia	Commonly reported^46^ Prevalence (PD): ~14%^46^	Rarely reported Prevalence: NA	Difficult
Misidentification Illusions	Commonly reported^15,43,46^ Prevalence (PD): ~20%^46^	Rarely reported^82^ Prevalence: NA	Difficult
Passage Hallucinations	Commonly reported^15,45,46^ Prevalence (PD): ~46%^15^	Rarely reported^49^ Prevalence: NA	Difficult
Presence Hallucinations	Commonly reported^15,45,46^ Prevalence (PD): ~25%^15^	Rarely reported^77,78^ Prevalence: NA	Difficult
Complex Hallucinations	Commonly reported^5,6,13,79,80^ Prevalence (PD): ~22%^5^	Commonly reported^20,49,82,83,85–87^ Prevalence (DMT): ~45%^86^	Possible

Table 1 outlines which hallucinatory phenomena are reasonable candidates for comparison between LBDs and SPs. While quantitative prevalence estimates are often available for LBDs, they remain sparse in the largely qualitative SP literature. To avoid artificial equivalence, the table presents both literature-based designations (“commonly,” “rarely,” or “not” reported) and quantitative estimates where available. These labels reflect either empirical strength or consistent anecdotal reporting, as supported by citations. A comparison is deemed “possible” when a phenomenon is commonly reported in both groups, “difficult” when common in one but rare in the other, and “not applicable” when absent from one group entirely.

## Cortical Function

### Differential Effects Across the Cortical Hierarchy

**Figure 1.**
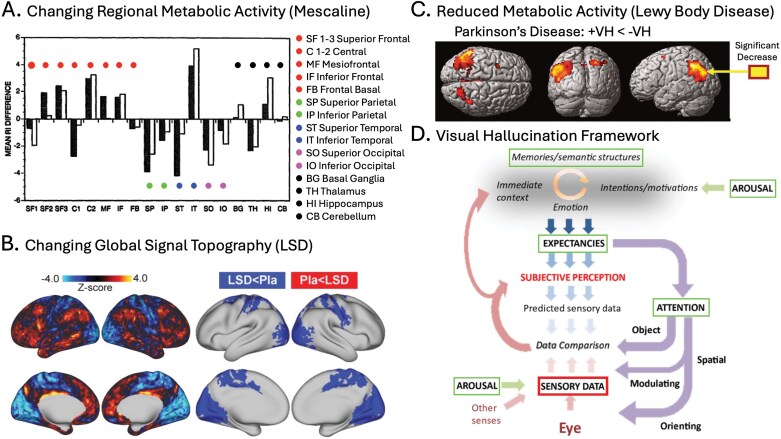
Differential effects across the cortical hierarchy. (A) From Hermle et al.,^94^ showing mescaline-induced changes in regional metabolic activity (SPECT-CBF) compared to baseline. Metabolic activity predominantly increases in frontal regions and decreases in parietal, temporal, and occipital areas, with the notable exception of inferior temporal cortex (IT). (B) From Preller et al.,^98^ showing LSD-induced changes in the fMRI global signal (GS) topography compared to placebo. The left images (unthresholded) depict GS modulation decreases in sensory-motor cortex (cold colors) and increases in the association cortex (warm colors). The right images (type-1 error corrected) depict significant GS reductions in sensory-motor cortex. (C) From Boecker et al.,^102^ showing reductions in regional metabolic rate of glucose consumption (FDG-PET) in the occipitotemporoparietal region of hallucinating PD patients (+ VH) compared with non-hallucinators (−VH). (D) From Collerton et al.,^113^ showing a framework for comparing across VH models. The image shows the range of cognitive, affective, perceptual, and sensory processes included in eight different models of VH. Different models posit that different combinations of top-down processes (ie, memories, expectancies, arousal, or attention) contribute to the production of VH, but all agree that degraded bottom-up sensory information (ie, sensory data) plays a key role in hallucinogenesis. *Source*: Data from Hermle et al., 1992, is reprinted from *Biological Psychiatry*, Vol. 32, Issue 11, with permission from Elsevier. Data from Boecker et al., 2007, is reprinted with permission from *JAMA Neurology*. Data from Preller et al., 2018, is reproduced under a CC BY 4.0 license from *eLife*. Data from Collerton et al., 2024, is reproduced under a CC BY 4.0 license from *Neuroscience and Biobehavioral Reviews*.

SPs consistently cause differential effects across the cortical hierarchy.^93^ The first modern neuroimaging study^94^ used single photon emission computed tomography (SPECT) to measure regional cerebral blood flow (CBF), showing mescaline-induced hyper-frontal and hypo-sensory metabolic changes (see Figure 1A for specific regions affected). Similar patterns have been observed using PET,^29,95,96^ MRI-derived CBF measures,^97^ and fMRI global signal topography Box 2 (see Figure 1B).^98^ These patterns of hyper- and hypo-activity align with a cortical divide between “intrinsic” (internal cognitive/affective) and “extrinsic” (sensory/perceptual) systems that are distinguished by gene expression^99^ and functional connectivity.^100^

Box 2. **Capturing the Onset, Persistence, and Resolution of Hallucinations**

Hallucinations occur episodically, not continuously. It is critical to identify features of the hallucination-prone brain when hallucinations are present and absent.^113^ Identifying features when hallucinations are absent, called “trait-based” research, could aid in developing diagnostic markers for early identification or management of hallucination susceptibility. “State-based” research emphasizes measuring neural signatures during hallucinatory episodes through hallucination capture experiments. Capture experiments have the potential to isolate dynamical neural processes responsible for transitions between veridical and hallucinatory perception, including the onset, persistence, and resolution of hallucinations.^113^

To date, hallucination capture experiments have identified a clear signature of *persistence*, potentially related to increased excitability in visual cortex: hyperactivity in category-selective visual cortices that match hallucinatory content. For example, hyperactivation in speech-related regions corresponds to auditory verbal hallucinations in schizophrenia,^149^ while hyperactivation of fusiform gyrus corresponds to face hallucinations in Charles Bonnet syndrome.^150^ Dujardin et al.^126^ reported similar effects during LBD hallucinations. While category-selective cortical activation serves as a marker of hallucinatory persistence, capturing signatures of the transition between veridical and hallucinatory perception is more challenging.^151,152^ Clinical hallucinations occur infrequently and unpredictably, making it difficult to systematically measure transient events like *onset* and *resolution*.^113^

SPs, on the other hand, reliably induce hallucinations that evolve dynamically, repeatedly emerging, persisting, and dissipating during the drug time course.^48^ While this dynamic context creates its own challenges, it also provides researchers with more opportunities to capture signatures of transitions between veridical and hallucinatory perception. Well-established effects of SP administration, such as visual activity suppression and cortical excitation, should be explored as candidate correlates of hallucinatory transitions.

Surprisingly, no SP hallucination capture experiments have been performed using modern neuroimaging methods. While many resting-state fMRI studies exist,^123^ including one exploring putative hallucinatory mechanisms,^153^ none track onsets or resolutions of specific episodes. Early SP researchers attempted to identify real-time EEG correlates of hallucinations,^154–156^ reporting that alpha-power suppression corresponds to hallucinatory periods. However, other early studies report significant individual variations in this effect.^157–162^ Modern SP-capture experiments could resolve these inconsistent observations.

In LBDs, studies report hypermetabolism in frontal regions^101^ and hypometabolism in primary and associative visual areas^70,102–106^. These effects distinguish LBD from non-hallucinatory neurodegenerative disorders (e.g., Alzheimer’s disease)^106^ and patients without hallucinations (Figure 1C)^102^. This differential activity may result from patterns of cortical neurodegeneration that are more pronounced in hallucinating patients. A recent mega-analysis^107^ found hallucinating Parkinson’s patients showed reduced cortical thickness in visual and frontal regions (i.e., occipitotemporal, parietal, and frontal cortex) and additionally reduced cortical surface area in occipital/occipitotemporal regions. Finally, resting-state fMRI dynamic causal modeling recently revealed that hallucinations in both LBDs^108^ and SPs^109^ were linked to reduced bottom-up and increased top-down effective connectivity. These findings align with theories suggesting that sensory deficits and associative hyper-activity contribute to hallucinations across contexts, including neurodegeneration^35, 110^, sensory deprivation^111^ and meditation^112^.

### Diverse Effects in Associative Cortex

Recently, Collerton et al.^113^ developed a framework (Figure 1D) to synthesize 8 leading models of VH, including several designed to model LBD hallucinations explicitly. Across the models, the contributions of top-down, associative processes vary considerably. According to different models, hallucinogenesis is attributed to various combinations of *attention* (Attention and Perception Deficit, APD; Activation-input Modulation,^114^ AIM; Attentional Networks,^115,116^ AN; Active Inference,^117^ AI; Thalamocortical Dysrhythmia Default Mode Network Decoupling,^118^ TDDMND), *expectation* (AN, AI, TDDMND), *memory* (AN, TDDMND, Reality Monitoring,^119^ RM), and *arousal* (AN), or suggest wide-spread network disfunction (ie, the Hodological model),^120^ or specify no role for top-down processing (ie, the Deafferentiation model).^121^

In SP research, similar model diversity exists. Divergent accounts of SP function may stem partly from an overemphasis on resting-state fMRI designs.^122,123^ During rest, SPs induce unpredictable mental states that can evolve rapidly, likely engaging different cortical networks. Combined with small sample sizes and other idiosyncratic factors, different studies observing different network activation patterns may have inspired different models. A biproduct of this diversity means evidence can be selectively compared, within and between LBD and SP studies, that aligns with or contradicts various models. For instance, a study supporting the Attentional Networks model^124^ found increased coupling between the default mode network (DMN) and the ventral attentional network (VAN) in hallucinating LBD patients. SPs can similarly induce DMN-VAN connectivity.^109,125^ However, the same study also found stronger connectivity within DMN nodes in hallucinators, a consistent finding in LBD research.^126–129^ This contrasts with SPs, which typically reduce within-network connectivity, including within the DMN, while increasing between-network connectivity.^130^ Reduced within-DMN connectivity is central to one leading theory of psychedelic function,^131^ though not all.^122^

Compared with resting state designs, task-based paradigms can target specific top-down functional networks, constraining comparisons across hallucinatory contexts. Moreover, established neurocognitive tasks that show reliable behavioral effects in one hallucinatory context could be validated in another. For example, perception of ambiguous stimuli^124^ and pareidolia^63,70^ correspond with LBD hallucinations and could be used to investigate attentional selection and expectation in SPs. In SPs, visual working memory dysfunction is well established^132,133^ and may overlap with findings on LBD hallucinations.^127,134^ Furthermore, task-based approaches are well placed for bridging clinical and preclinical research.^135^

The diversity of hallucinatory models raises the additional question of whether VH across contexts and types (eg, clinical/pharmacological or metamorphopsias/entities) share a common mechanism or emerge from multiple independent pathways. Collerton et al.^113^ propose a middle ground between these two extremes that highlights a key point in the hallucinogenic process: the comparison of degraded sensory data with internal expectations. Evidence that a *specific* top-down, associative process is contributing to hallucinogenesis (eg, one generating expectations via memory or comparisons via attention) may vary depending on the specific hallucination context or type. However, across hallucinatory contexts and phenomena, there is evidence that degraded bottom-up, sensory information plays a key role in hallucinogenesis.

### Reduced Visually Evoked Activity

Degraded visual information is a unifying feature of every model reviewed by Collerton et al. (2024). The failure of the visual system to encode and/or represent high-fidelity sensory information may drive various top-down processes to “fill the gaps,” resulting in hallucinatory experience. In LBDs, visual dysfunction is well established.^136^ It can result from a range of ocular and visual disturbances^137^ and corresponds with minor hallucinations more so than complex hallucinations.^138^ Multiple genetic markers have been linked to these visual deficits, as well as to reductions of both metabolic activity and blood flow in posterior brain regions in these patients (see Weil et al.^65^ for a comprehensive review).

**Figure 2.**
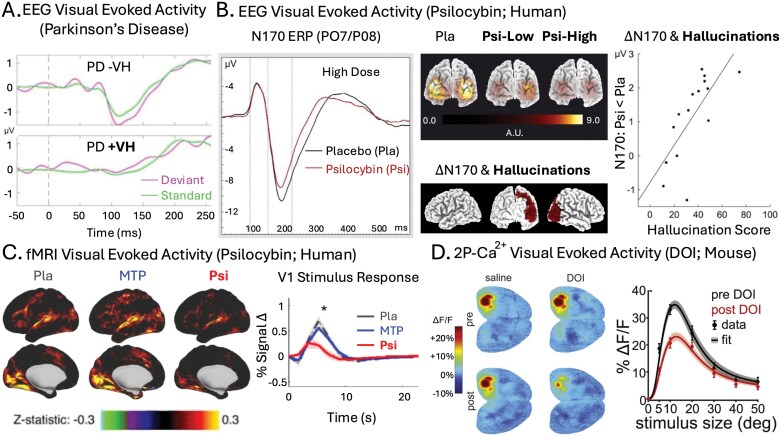
Reduced Stimulus Evoked Response. (A) From Vignando et al.,^141^ showing averaged EEG visually evoked activity in hallucinating (+VH) and non-hallucinating (−VH) Parkinson’s disease (PD) patients evoked during a mismatched negativity task (MMN). In +VH, no negative evoked response potential (ERP) is apparent, while one is clearly evident in −VH for both trial types. (B) From Kometer et al.,^142,143^ showing averaged EEG visually evoked activity during psilocybin (Psi) and placebo (pla) administration. Left panel depicts the suppression of N170 evoked response potentials (ERP) during a high-dose Psi condition. Middle panels show that this suppression is dose-dependent (Psi-Low and Psi-High) and localized to posterior cortex (top images), and that reduced activity in right-posterior cortex correlates significantly with measures of SP-induced hallucinations (bottom images). Right panel shows a similar correlation between hallucination scores and N170 suppression from a later study, where 5-HT_2A_R blockade was shown to block both Psi-induced N170 suppression and hallucinations. (C) From Siegel et al.,^125^ showing fMRI visual evoked activity during Psi, Pla, and methylphenidate (MTP) administration in humans. Left images depict cortex-wide effects of visual stimulation and right image depicts time course of average voxel activity in V1. Both demonstrate suppressed visual activity during Psi compared to control conditions. (D) From Michaiel et al.,^144^ showing widefield 2P-Ca^2+^ visual evoked activity during DOI and saline (Sal) administration in mice. Left images depicts cortex-wide effects of visual stimulation pre and post, with DOI suppressing visual activity in V1 post administration. Right image depicts stimulus-size tuning curves for individual V1 neurons (layer II/III), with DOI again showing a suppressive effect. *Source*: Data from Vignando et al., 2022, and Siegel et al., 2024, are reproduced under a CC BY 4.0 license. Data from Kometer et al., 2011, is reprinted from *Biological Psychiatry*, Vol. 69, Issue 5, with permission from Elsevier. Data from Kometer et al., 2013, is adapted from *Journal of Neuroscience* under a CC-BY-NC-SA 3.0 license. Data from Michaiel et al., 2019, is reprinted from *Cell Reports*, Vol. 26, Issue 13, with permission from Elsevier.

One functional signature of degraded visual processing in LBDs is reduced stimulus-driven activity in visual regions. fMRI studies have linked LBD hallucinations to reduced activity in occipital^139^ and temporal visual cortices,^140^ as well as higher-order visual regions like the superior parietal lobule and the frontal eye fields.^124^ Multiple studies have found reduced activation in the higher-order motion area MT in LBD patients.^59–62,124^ Recently, Vignando et al.^141^ show that the magnitude EEG evoked response potentials (ERPs) in hallucinating LBD patients are notably degraded, compared with non-hallucinators (see Figure 2A).

SPs also reliably reduce stimulus-evoked activity in visual regions. In multiple EEG studies,^142,143,145,146^ SP administration suppressed the N170 ERP. The N170 is a negative potential that occurs ~170 ms after stimulus onset and is typically associated with the perception of complex visual objects, faces in particular.^147^ Crucially, this signature of visual suppression correlated significantly with subjective measures of VH (Figure 2B) and is reversed by blocking the 5-HT_2A_ receptor (see the Serotonin Receptor section for more details).^143^ Additionally, fMRI evidence of SP-reduced stimulus-driven activity has been observed in higher-order visual regions (i.e., fusiform gyrus and inferior temporal gyrus),^148^ and observations with both human fMRI^125^ and mouse two-photon calcium imaging (2P-Ca^2+^)^144^ studies find SPs reduce stimulus-driven activity in primary visual cortex (V1; Figure 2B, C). While there is a clear correlation between SP-induced visual suppression and hallucinations, it remains to be shown whether this suppression plays a direct, causal role in SP hallucinogenesis (see Box 2).

### Hyperexcitability in Visual Cortex

Perhaps surprisingly, hyperexcitability in the visual cortex, in addition to reduced sensory activation, is associated with VH in both LBDs and SPs. In LBDs, multiple lines of evidence link hallucinations to visual cortex hyperexcitability. Taylor et al.^163^ found hallucination severity correlated with phosphene thresholds in LBD patients. Hallucinators experienced more complex percepts, suggesting a link between excitability and hallucinations. In a follow-up fMRI study,^164^ phosphene thresholds positively correlated with BOLD activity in visual areas in healthy controls but negatively in LBD patients, indicating a loss of inhibitory control. Reduced GABAergic inhibition in hallucinating patients, observed in postmortem studies^165^ and magnetic resonance spectroscopy,^59^ further supports increased excitability. Similar changes occur following visual deprivation, where lower GABA levels shift the excitatory/inhibitory (E/I) balance toward excitation.^166^

In SPs, empirical evidence of hyperexcitability in the visual cortex is primarily derived from suppressed oscillatory power in the alpha bandwidth (8-13Hz) in EEG/MEG signals. Alpha power modulates cortical excitation^167^ and perceptual sensitivity.^168^ Alpha suppression was observed in the very first psychedelic neuropharmacology study^154^ and remains a robust correlate of SP administration.^143,169–176^ Recently, Muthukumaraswami and Liley^177^ showed SP-induced alpha suppression correlates with a proxy for E/I balance (i.e., flattening of the 1/f slope in the 30-50Hz range),^178^ indicating a shift toward greater excitation. Whether such excitatory shifts directly trigger hallucinations, cause their persistence, simply increase their likelihood, or are an entirely unrelated phenomenon remains unknown. Addressing this requires experiments measuring SP neural signatures during the onset, persistence, and resolution of hallucinatory events (see Box 2).

Psilocybin has also been shown to amplify the P100 ERP, a positive potential ~100 ms after stimulus onset that is thought to reflect early processing of visual input.^179^ Across the four studies that found N170 suppression (reviewed above), there was disagreement regarding effects on the P100. Two studies employing higher-order stimuli (i.e., faces) reported no effect on the P100,^145,146^ while two studies using lower-order stimuli (i.e., Kanizsa stimuli), reported marginal P100 facilitation constrained to medial-occipital electrodes.^142,143^ This stimulus selective facilitatory effect suggests that 5-HT_2A_R activation may be excititory earlier in the visual hierarchy and suppressive downstream. Crucially, this is at odds with observations from animal electrophisiology in primary visual cortex,^144,180,181^ indicating that more work is needed to resolve this discrepancy.

It is important to note that hyperexcitability in frontal cortex also likely contributes to LBD and SP hallucinogenesis via feedback circuitry.^30,108,109^ Such feedback may drive the diverse set of top-down mechanisms proposed by the models discussed above. While it is crucial to understand such contributions, in this review we suggest that bottom-up signatures may offer clearer targets for cross-group comparison.

### Balancing Suppression and Excitation?

Of the eight models reviewed in Collerton et al. (2024), the Deafferentation Model most directly addresses the paradoxical link between degraded sensory input and visual hyperexcitability. This model was proposed to explain hallucinations in eye disease (ie, Charles Bonnet Syndrome) and sensory deprivation,^64,121^ and suggests that visual deprivation disconnects ascending inputs from upstream cortices, causing compensatory hyperexcitation. Recent work links this disconnection process to destabilized homeostatic plasticity and a shift in cortical E/I balance toward excitation.^182,183^ As SPs cause both reduced visual activation and cortical excitability, they may be useful for investigating links between disturbed homeostatic processes and hallucinogenesis.

Intriguingly, dysregulation of related homeostatic mechanisms contribute to visual plasticity during developmental critical periods.^184,185^ Critical period plasticity has been implicated in the effects of SP-administration,^186–188^ and recent theoretical work suggests CP plasticity plays a role in Hallucination Persisting Perception Disorder.^189^ Thus, investigating SP-induced visual suppression and E/I modulation might reveal correspondences between homeostatic dysregulation,^182^ critical period plasticity,^184^ and hallucinogenesis.^183^

### Serotonin Receptors

#### 5-HT_2A_R & 5-HT_1A_R Comodulate Hallucinations

Activation of the 5-HT_2A_R receptor is essential for SP-induced hallucinogenesis. Vollenweider et al.^29^ showed that blocking 5-HT_2A_R with ketanserin dose-dependently reduced and eventually abolished psilocybin’s hallucinogenic effects. Other SPs show similar modulation.^26,28^ The 5-HT_1A_R receptor also plays a role. Its blockade intensifies DMT hallucinations^27^ and its activation attenuates the hallucinatory effects of psilocybin.^25^ These findings suggest 5-HT_2A_R and 5-HT_1A_R co-modulate SP-induced hallucinations.

Parkinsonian disorders are generally associated with loss of dopaminergic function and LBD hallucinations are closely associated with cholinergic modulation (see Box 3). However, there is mounting pharmacological evidence that interfering with 5-HT_2A_R activity reduces hallucinations and other symptoms of psychosis in LBDs.^30^ The 5-HT_2A_R antagonist clozapine and inverse agonist pimavanserin have both proven effective at treating hallucinations in patients with Parkinson’s disease psychosis (PDP).^31–33^ Pimavanserin was recently approved for treatment of PDP by the United States Food and Drug Administration.^208^

Box 3. **Complex Neuromodulator Interactions Contribute to Hallucinogenesis**

This review focuses on the link between the serotonergic system and VH, due to its clear role in SP hallucinations.^29^ However, serotonergic, dopaminergic, and cholinergic systems are closely intertwined in LBDs. Dopaminergic neurodegeneration is a hallmark of LBDs,^190–192^ while degeneration in cholinergic output regions, such as the pedunculopontine nucleus^193^ and basal forebrain,^194^ is linked to LBD hallucinations. Dopaminergic medications can worsen LBD hallucinations,^195–197^ whereas cholinergic medications can improve them.^198^ Finally, Perry et al.^199^ found that hallucinating DLB patients showed severe cholinergic dysfunction but stable dopaminergic and serotonergic function, while non-hallucinators exhibited the opposite pattern.

According to predictive coding frameworks, cholinergic and dopaminergic systems may differentially modulate bottom-up and top-down factors. Cholinergic function enhances sensory precision by up-weighting prediction errors in response to unexpected sensory data, prompting further exploration and updating of internal models (ie, perception).^200–202^ Cholinergic *dysfunction* then, degrades sensory precision and disrupts perceptual updating, ultimately discounting sensory data (ie, bottom-up factors). This failure to reconcile sensory evidence with faulty internal models (ie, hallucinations) may contribute to hallucinatory *persistence*.^113^ In contrast, dopaminergic processes modulate high-level priors through prediction errors.^203,204^ Hyperactivation of this system boosts confidence in high-level priors, shifting influence over perceptual updating away from sensory data toward amplified expectations (ie, top-down factors). Fluctuating confidence in these expectations may influence hallucinatory *transitions.*

Teasing apart bottom-up and top-down influences of interacting neuromodulatory systems is a complex goal. Making progress requires aligning task-based and comparative neuropharmacology paradigms across clinical and preclinical experiments.^135^ Some efforts along these lines have been made. For instance, Schmack et al.^204^ used human psychophysics, pharmacological manipulation, and rodent optogenetics to show that dopaminergic neurons in mice causally increase high-confidence false alarms (ie, “hallucination-like” percepts) in an auditory signal detection task. Anticholinergic drugs that reliably induce hallucinations (eg, scopolamine)^205^ also increase false alarms.^206^ It remains unclear whether SPs likewise modulate false-alarm rates.^207^ Cross-species studies that combine dopaminergic, cholinergic, and serotonergic manipulations in signal detection paradigms or other neurocognitive tasks would significantly clarify how these systems contribute to hallucinatory mechanisms.

Of particular relevance to this review, SP administration has proved useful for exploring the effect of pimavanserin-modulated 5-HT_2A_R activation in animal models of Parkinson’s disease. McFarland et al.^209^ employed the head twitch response (HTR), a motor action and key assay of 5-HT_2A_R activation,^210,211^ which may index hallucinations in mice. Parkinsonian mice exhibited more spontaneous HTRs, possibly signifying increased LBD-induced hallucinations. They also showed more HTR following SP-administration. Pimavanserin reduced both spontaneous and SP-induced HTRs.

#### 5-HT_2A_R Upregulation in LBDs

Why is the serotonin system implicated in LBD hallucinations? There is evidence that the 5-HT_2A_R is upregulated in hallucinating patients.^212,213^ In a postmortem study of PD patients, Huot et al.^214^ reported nearly 50% more 5-HT_2A_R binding in the inferolateral temporal cortex in patients who experienced VH compared to those that did not. In a PET study, Ballanger et al.^215^ observed increased 5-HT_2A_R binding in the ventral visual pathway and in frontal cortices among hallucinating PD patients.

5-HT_2A_R upregulation is also observed in rodent models of PD.^216–220^ There is evidence this effect is a homeostatic response to the loss of dopaminergic function in these animals. Li et al.^221^ found that reduced dopaminergic function in a PD animal model led to greater serotonergic activity and 5-HT_2A_R upregulation. Stimulating 5-HT_2A_R with the psychedelic DOI rescued motor dysfunction, while blocking it worsened symptoms. These findings suggest 5-HT_2A_R upregulation may compensate for dopaminergic loss in PD, aiding motor function. Thus, LBD hallucinations may result from complex interactions between dopaminergic and serotonergic systems (see Box 3).

#### Excitation in Frontal Cortex

It has been proposed that hyperexcitability in the medial prefrontal cortex (mPFC) contributes to LBD hallucinations.^30^ PD animal models with surgically-induced dopaminergic dysfunction show increased mPFC excitability.^222,223^ Moreover, in the same animals, the excitatory effect of SP administration in mPFC is suppressed. This further suggests that complex dopaminergic and serotonergic interactions are involved in LBD hallucinogenesis (see Box 3).

The effects of SPs are also linked to hyperexcitability in mPFC.^131,224,225^ In animal models, both DOI^226,227^ and 5-MeO-DMT^228^ cause an overall excitatory effect in mPFC. This effect results from changes in E/I balance in frontal cortex, which is comodulated by the 5-HT_2A_R and 5-HT_1A_R receptors.^229,230^ Such shifts in E/I balance have long been thought to contribute to hallucinogenesis in both psychosis and psychedelics.^34,231–233^

#### Suppression in Visual Cortex

Frontal excitability is only half the story, however. Emerging evidence suggests that serotonin primarily suppresses activity in the visual cortex. In a recent optogenetic experiment, Azimi et al.^180^ demonstrated that selectively driving either the 5-HT_1A_R or 5-HT_2A_R suppresses activity in mouse V1. Driving 5-HT_1A_R selectively suppresses spontaneous (but not visually evoked) activity, while driving the 5-HT_2A_R selectively suppresses visually evoked (but not spontaneous) activity. This impressive double dissociation is at least partially consistent with the effects of SPs in mice. Administering DOI, a 5-HT_2A_R (but not 5-HT_1A_R) agonist, suppresses visually evoked (but not spontaneous) activity in mouse V1 (see Figure 2D).^144^

**Figure 3.**
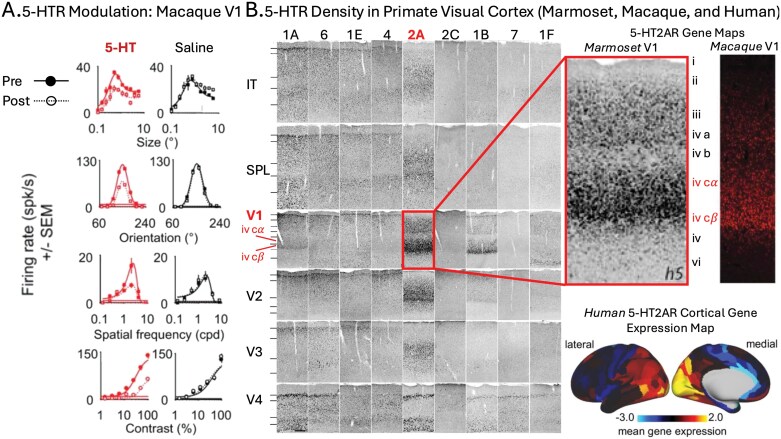
Serotonin Receptor (5-HTR) Modulation and Location in Primate Visual Cortex. (A) From Seillier et al.,^181^ showing effects of 5-HT iontophoresis in macaque primary visual cortex (V1) during visual stimulation. Compared with saline (NaCl, right panel column), 5-HT administration (dashed-open lines, left panel column) systematically decreased response amplitude of tuning curves for size, orientation, spatial frequency, and contrast stimuli. (B) From Shukla et al.,^234^ Watakabe et al.,^235^ and Preller et al.,^98^ showing 5-HTR Density in visual cortex of marmoset, macaque, and human, respectively. *Left panel:* Distribution of serotonin receptors derived from in situ hybridization (ISH) across marmoset cortical areas (V1; extrastriate cortex: V2-4; superior parietal lobule: SPL; inferior temporal: IT), showing dense gene expression of 5-HT_2A_R receptors in geniculorecipient layers IVc α/βof V1. *Upper right panels:* A magnified view of the marmoset V1 5-HT_2A_R receptor distribution (left) compared side-by-side with macaque V1 receptor images (ISH), illustrating a similar concentration of 5-HT_2A_R receptors localized to V1 input layers IVc α/β . *Lower right panel:* Human 5-HT_2A_R gene expression map derived from microarray transcriptional profiling (courtesy of the Allen Human Brain Atlas), showing the cortical distribution of 5-HT_2A_R mRNA expression. The map highlights the dense 5-HT_2A_R expression in human primary visual cortex. *Source*: Data from Seillier et al., 2017, and Shukla et al., 2015, are reproduced under a CC BY 4.0 license. Data from Watakabe et al., 2009, is reproduced under a CC BY-NC 2.0 UK license. Data from Preller et al., 2018, is reproduced under a CC BY 4.0 license from *eLife*.

Seillier et al.^181^ showed that serotonin suppresses visually evoked activity in *primate* V1 (Figure 3A) in a manner similar to 5-HT_2A_R-dependent visual suppression in mouse V1 (Figure 2D). Although the observation made by Seillier et al. does not directly implicate the 5-HT_2A_R in primate visual suppression, it is worth noting that this receptor is especially densely expressed in primate V1. More narrowly, 5-HT_2A_R expression is highly localized to *layer IVc* of the primary visual cortex: the input layer carrying retinal information from thalamus to cortex (Figure 3B). This location at the base of the visual cortical hierarchy is uniquely suited for modulating processes throughout the visual system.^236^

Two questions present themselves: (A) Does 5-HT_2A_R activity suppress thalamic input to layer IVc of V1 in primates? (B) Does this putative suppression causally contribute to SP hallucinations? In human research, SP-induced visual suppression and hallucinations are clearly correlated: blocking the 5-HT_2A_R blocks both effects (Figure 2B).^143^ However, this could mean they share a common cause (eg, hyperexcitability), rather than visual suppression directly contributing to hallucinations. Addressing points A and B would determine whether visual degradation plays a mechanistic role in SP hallucinogenesis.^113^

While 5-HT_2A_R-driven visual suppression may contribute to SP-induced hallucinations directly, its role in LBD hallucinations is less clear. One possibility is that *chronic* serotonergic suppression influences cortical degeneration in visual regions, leading to LBD hallucinogenesis. Vignando et al.^107^ found that 5-HT_2A_R /5-HT_1A_R receptor distributions correlate with hallucination-specific cortical degeneration patterns, distinct from those linked to dopamine. In a potentially related finding, Zarkali et al.^237^ reported that the distribution of 5-HT_2A_R is linked to altered dynamic transitions between integrated and segregated functional connectivity states that are characteristic of hallucinating PD patients. The specific interactions between 5-HT_2A_R topography, functional connectivity states, and patterns of cortical degeneration in hallucinating patients remain unclear. Future studies using both neuroimaging with patients and Parkinsonian model animals should explore how serotonin’s suppressive effects in visual regions might contribute to these structural and functional patterns across the cortical visual hierarchy.

Box 4. **Future Directions**

**Phenomenology**

[1] Psychophysical experiments on visual distortions common to LBDs and SPs could reveal shared neural mechanisms.
[2] Interviewing LBD patients who experienced SP-induced hallucinations prior to their diagnosis could clarify phenomenological similarities and the role of expectation in shaping content.
[3] Quantifying hallucination subtypes in SPs could clarify their prevalence and improve cross-group comparisons.

**Cortical Function**

[1] Using consistent neurocognitive tasks (eg, signal detection, ambiguous stimuli, visual working memory) across hallucinatory contexts could pinpoint top-down processes driving specific hallucinations.
[2] Probing suppressed visual evoked activity and E/I balance shifts could illuminate the role of homeostatic plasticity in hallucinogenesis.
[3] SP capture experiments could isolate hallucinatory onset and resolution signatures.

**Serotonin Receptors**

[1] Combining neuropharmacology with neurocognitive tasks in cross-species studies could clarify serotonergic, dopaminergic, and cholinergic roles in hallucinations.
[2] Primate studies could test if 5-HT_2A_R activity suppresses thalamic input to V1.
[3] Parkinsonian animal models could reveal whether serotonergic visual suppression contributes to LBD cortical neurodegeneration.

### Conclusion

In this review, we compared visual hallucinatory phenomena in LBDs and SPs, proposing that focused research on shared hallucinatory features may help identify common neural mechanisms. We discussed three cortical signatures found in both LBDs and SPs: hypo-/hyper-activation in sensory/associative regions, reduced visual evoked activity, and a shift in E/I balance toward excitation. We highlight the role of 5-HT_2A_R /5-HT_1A_R in hallucinations, suggesting that 5-HT_2A_R --driven suppression at the base of the cortical visual hierarchy is a potential hallucinogenic mechanism. In SPs, transient visual suppression may trigger compensatory excitability in the associative visual cortex or higher-order regions, contributing to hallucinogenesis. In LBDs, chronic suppression may cause visual-hierarchy degeneration, fostering persistent hallucination proneness. Leveraging SP-induced hallucinations, task-based paradigms, and comparative neuropharmacology across populations and species could reveal shared mechanisms, driving theoretical and clinical advances. Insights into these mechanisms may clarify the fundamental sensory effects of SP administration^236^ and guide novel interventions for LBD hallucination, potentially improving clinical management and patient quality of life.

## References

[1] Sacks O. Hallucinations. Pan Macmillan; 2012.

[2] McKeith I, Mintzer J, Aarsland D, et al; International Psychogeriatric Association Expert Meeting on DLB. Dementia with Lewy bodies. Lancet Neurol. 2004;3:19–28. https://doi.org/10.1016/S1474-4422(03)00619-7 14693108

[3] Wakabayashi K, Tanji K, Odagiri S, Miki Y, Mori F, Takahashi H. The Lewy body in Parkinson’s disease and related neurodegenerative disorders. Mol Neurobiol. 2013;47:495–508. https://doi.org/10.1007/s12035-012-8280-y 22622968

[4] D’Antonio F, Boccia M, Di Vita A, et al Visual hallucinations in Lewy body disease: pathophysiological insights from phenomenology. J Neurol. 2022;269:3636–3652. https://doi.org/10.1007/s00415-022-10983-6 35099586 PMC9217885

[5] Fénelon G, Mahieux F, Huon R, Ziégler M. Hallucinations in Parkinson’s disease: prevalence, phenomenology and risk factors. Brain. 2000;123 ( Pt 4):733–745. https://doi.org/10.1093/brain/123.4.733 10734005

[6] Rabey JM. Hallucinations and psychosis in Parkinson’s disease. Parkinsonism Relat Disord. 2009;15:S105–S110. https://doi.org/10.1016/S1353-8020(09)70846-6 20123547

[7] Kyle K, Bronstein JM. Treatment of psychosis in Parkinson’s disease and dementia with Lewy Bodies: A review. Parkinsonism Related Disord. 2020;75:55–62. https://doi.org/10.1016/j.parkreldis.2020.05.026 32480308

[8] Pagonabarraga J, Bejr-Kasem H, Martinez-Horta S, Kulisevsky J. Parkinson disease psychosis: from phenomenology to neurobiological mechanisms. Nat Rev Neurol. 2024;20:135–150. https://doi.org/10.1038/s41582-023-00918-8 38225264

[9] Friedman JH. Dementia with Lewy bodies and Parkinson disease dementia: it is the same disease! Parkinsonism Related Disord. 2018;46:S6–S9. https://doi.org/10.1016/j.parkreldis.2017.07.013 28756177

[10] Ravina B, Marder K, Fernandez HH, et al Diagnostic criteria for psychosis in Parkinson’s disease: Report of an NINDS, NIMH work group. Movement Disord. 2007;22:1061–1068. https://doi.org/10.1002/mds.21382 17266092

[11] Bejr-kasem H, Sampedro F, Marín-Lahoz J, Martínez-Horta S, Pagonabarraga J, Kulisevsky J. Minor hallucinations reflect early gray matter loss and predict subjective cognitive decline in Parkinson’s disease. Eur J Neurol. 2021;28:438–447. https://doi.org/10.1111/ene.14576 33032389

[12] Fénelon G, Soulas T, De Langavant LC, Trinkler I, Bachoud-Lévi AC. Feeling of presence in Parkinson’s disease. J Neurol, Neurosurg Psychiat. 2011;82:1219–1224. https://doi.org/10.1136/jnnp.2010.234799 21551471 PMC3382202

[13] ffytche DH, Creese B, Politis M, et al The psychosis spectrum in Parkinson disease. Nat Rev Neurol. 2017;13:81–95. https://doi.org/10.1038/nrneurol.2016.200 28106066 PMC5656278

[14] Goetz CG, Fan W, Leurgans S, Bernard B, Stebbins GT. The malignant course of “benign hallucinations” in Parkinson disease. Arch Neurol. 2006;63:713–716. https://doi.org/10.1001/archneur.63.5.713 16682540

[15] Lenka A, Pagonabarraga J, Pal PK, Bejr-Kasem H, Kulisvesky J. Minor hallucinations in Parkinson disease. Neurology. 2019;93:259–266. https://doi.org/10.1212/WNL.0000000000007913 31289146 PMC6709995

[16] Capouch SD, Farlow MR, Brosch JR. A review of dementia with Lewy Bodies’ impact, diagnostic criteria and treatment. Neurol Ther. 2018;7:249–263. https://doi.org/10.1007/s40120-018-0104-1 29987534 PMC6283803

[17] Factor SA, Feustel PJ, Friedman JH, et al; Parkinson Study Group. Longitudinal outcome of Parkinson’s disease patients with psychosis. Neurology. 2003;60:1756–1761. https://doi.org/10.1212/01.WNL.0000068010.82167.CF 12796526

[18] Goetz CG, Stebbins GT. Mortality and hallucinations in nursing home patients with advanced Parkinson’s disease. Neurology. 1995;45:669–671. https://doi.org/10.1212/WNL.45.4.669 7723953

[19] Schrag A, Hovris A, Morley D, Quinn N, Jahanshahi M. Caregiver-burden in parkinson’s disease is closely associated with psychiatric symptoms, falls, and disability. Parkinsonism Related Disord. 2006;12:35–41. https://doi.org/10.1016/j.parkreldis.2005.06.011 16271496

[20] Kometer M, Vollenweider FX. Serotonergic hallucinogen-induced visual perceptual alterations. In: Halberstadt AL, Vollenweider FX, Nichols DE, eds. Behavioral Neurobiology of Psychedelic Drugs. Springer; 2018:257–282. https://doi.org/10.1007/7854_2016_461 27900674

[21] Onofrj M, Russo M, Delli Pizzi S, et al The central role of the Thalamus in psychosis, lessons from neurodegenerative diseases and psychedelics. Transl Psychiatry. 2023;13:384. https://doi.org/10.1038/s41398-023-02691-0 38092757 PMC10719401

[22] Russo M, Carrarini C, Dono F, et al The pharmacology of visual hallucinations in synucleinopathies. Front Pharmacol. 2019;10:1379. https://doi.org/10.3389/fphar.2019.01379 31920635 PMC6913661

[23] Kwan AC, Olson DE, Preller KH, Roth BL. The neural basis of psychedelic action. Nat Neurosci. 2022;25:1407–1419. https://doi.org/10.1038/s41593-022-01177-4 36280799 PMC9641582

[24] Ballentine G, Friedman SF, Bzdok D. Trips and neurotransmitters: discovering principled patterns across 6850 hallucinogenic experiences. Sci Adv. 2022;8:eabl6989. https://doi.org/10.1126/sciadv.abl6989 35294242 PMC8926331

[25] Pokorny T, Preller KH, Kraehenmann R, Vollenweider FX. Modulatory effect of the 5-HT1A agonist buspirone and the mixed non-hallucinogenic 5-HT1A/2A agonist ergotamine on psilocybin-induced psychedelic experience. Eur Neuropsychopharmacol. 2016;26:756–766. https://doi.org/10.1016/j.euroneuro.2016.01.005 26875114

[26] Preller KH, Herdener M, Pokorny T, et al The fabric of meaning and subjective effects in LSD-induced states depend on serotonin 2A receptor activation. Curr Biol. 2017;27:451–457. https://doi.org/10.1016/j.cub.2016.12.030 28132813

[27] Strassman RJ. Human psychopharmacology of N,N-dimethyltryptamine. Behav Brain Res. 1995;73:121–124. https://doi.org/10.1016/0166-4328(96)00081-2 8788488

[28] Valle M, Maqueda AE, Rabella M, et al Inhibition of alpha oscillations through serotonin-2A receptor activation underlies the visual effects of ayahuasca in humans. Eur Neuropsychopharmacol.. 2016;26:1161–1175. https://doi.org/10.1016/j.euroneuro.2016.03.012 27039035

[29] Vollenweider FX, Vollenweider-Scherpenhuyzen MFI, Bäbler A, Vogel H, Hell D. Psilocybin induces schizophrenia-like psychosis in humans via a serotonin-2 agonist action. Neuroreport. 1998;9:3897–3902. 9875725 10.1097/00001756-199812010-00024

[30] Burstein ES. Relevance of 5-HT2A Receptor modulation of pyramidal cell excitability for dementia-related psychosis: implications for pharmacotherapy. CNS Drugs. 2021;35:727–741. https://doi.org/10.1007/s40263-021-00836-7 34224112 PMC8310514

[31] Meltzer HY, Kennedy J, Dai J, Parsa M, Riley D. Plasma clozapine levels and the treatment of L-DOPA-induced psychosis in Parkinson’s disease. a high potency effect of clozapine. Neuropsychopharmacology. 1995;12:39–45. https://doi.org/10.1016/0893-133X(94)00060-D 7766285

[32] Cummings J, Isaacson S, Mills R, et al Pimavanserin for patients with Parkinson’s disease psychosis: a randomised, placebo-controlled phase 3 trial. Lancet (London, England). 2014;383:533–540. https://doi.org/10.1016/S0140-6736(13)62106-6 24183563

[33] Hacksell U, Burstein ES, McFarland K, Mills RG, Williams H. On the discovery and development of pimavanserin: a novel drug candidate for Parkinson’s psychosis. Neurochem Res. 2014;39:2008–2017. https://doi.org/10.1007/s11064-014-1293-3 24682754 PMC4172996

[34] Leptourgos P, Fortier-Davy M, Carhart-Harris R, et al Hallucinations under psychedelics and in the schizophrenia spectrum: an interdisciplinary and multiscale comparison. Schizophr Bull. 2020;46:1396–1408. https://doi.org/10.1093/schbul/sbaa117 32944778 PMC7707069

[35] Waters F, Collerton D, ffytche DH, et al Visual hallucinations in the psychosis spectrum and comparative information from neurodegenerative disorders and eye disease. Schizophr Bull. 2014;40:S233–S245. https://doi.org/10.1093/schbul/sbu036 24936084 PMC4141306

[36] Montagnese M, Leptourgos P, Fernyhough C, et al A review of multimodal hallucinations: categorization, assessment, theoretical perspectives, and clinical recommendations. Schizophr Bull. 2021;47:237–248. https://doi.org/10.1093/schbul/sbaa101 32772114 PMC7825001

[37] Blom JD. A Dictionary of Hallucinations. New York: Springer; 2010. https://doi.org/10.1007/978-1-4419-1223-7

[38] Beze S, Castellani L, Pereira B, Chiambaretta F, Durif F, Marques A. Two-year longitudinal follow-up of visual illusions and hallucinations in Parkinson’s disease. J Neurol. 2022;269:4546–4554. https://doi.org/10.1007/s00415-022-11074-2 35296961

[39] Jucevičiūtė N, Balnytė R, Laucius O. Exploring the spectrum of visual illusions and other minor hallucinations in patients with parkinson’s disease in Lithuania. Medicina (Kaunas). 2024;60:606. https://doi.org/10.3390/medicina60040606 38674252 PMC11051838

[40] Marques A, Beze S, Pereira B, et al Visual hallucinations and illusions in Parkinson’s disease: the role of ocular pathology. J Neurol. 2020;267:2829–2841. https://doi.org/10.1007/s00415-020-09925-x 32447550

[41] Marques A, Taylor NL, Roquet D, et al Structural and functional correlates of hallucinations and illusions in parkinson’s disease. Journal of Parkinson’s Disease. 2022;12:397–409. https://doi.org/10.3233/JPD-212838 34744050

[42] Nishio Y, Yokoi K, Uchiyama M, et al Deconstructing psychosis and misperception symptoms in Parkinson’s disease. J Neurol Neurosurg Psychiatry. 2017;88:722–729. https://doi.org/10.1136/jnnp-2017-315741 28600444

[43] Nishio Y, Yokoi K, Hirayama K, et al Defining visual illusions in Parkinson’s disease: Kinetopsia and object misidentification illusions. Parkinsonism Related Disord. 2018;55:111–116. https://doi.org/10.1016/j.parkreldis.2018.05.023 29891431

[44] Sasaki C, Yokoi K, Takahashi H, et al Visual illusions in Parkinson’s disease: an interview survey of symptomatology. Psychogeriatrics. 2022;22:38–48. https://doi.org/10.1111/psyg.12771 34617361 PMC9293438

[45] Wang Y, Li D, Chen Y, et al Clinical features of minor hallucinations in different phenotypes of Parkinson’s disease: a cross-sectional study. Front Neurol. 2023;14:1158188. https://doi.org/10.3389/fneur.2023.1158188 37034082 PMC10079986

[46] Zhong M, Gu R, Zhu S, et al Prevalence and risk factors for minor hallucinations in patients with Parkinson’s disease. Behav Neurol. 2021;2021:3469706. https://doi.org/10.1155/2021/3469706 34646400 PMC8505047

[47] Díaz JL. Sacred plants and visionary consciousness Sci. Phenom Cogn. 2010;9:159–170. https://doi.org/10.1007/s11097-010-9157-z

[48] Jarvik M. E. S RK. Drug-induced hallucinations in animals and man. Hallucinations: Behav, Exper theory. 1975;81:161.

[49] Noah S, Shen M, Erowid E, Erowid F, Silver M. A novel method for quantitative analysis of subjective experience reports: application to psychedelic visual experiences. Front Psychol. 2024;15:1397064. https://doi.org/10.3389/fpsyg.2024.1397064 39712538 PMC11663017

[50] Hollister LE, Hartman AM. Mescaline, lysergic acid diethylamide and psilocybin: comparison of clinical syndromes, effects on color perception and biochemical measures. Compr Psychiatry. 1962;3:235–242. https://doi.org/10.1016/S0010-440X(62)80024-8 13908449

[51] Fischer R, Hill RM, Warshay D. Effects of the psychodysleptic drug psilocybin on visual perception. changes in brightness preference. Experientia. 1969;25:166–169. 4389138 10.1007/BF01899102

[52] Hill RM, Fisher R, Warshay D. Effects of ecitatory and tranquilizing drugs on visual perception. spatial distortion thresholds. Experientia. 1969;25:171–172. 5786095 10.1007/BF01899105

[53] Shaffer JH, Hill RH, Fischer R. Psychophysics of psilocybin and delta-9-tetrahydrocannabinol. Agents Actions. 1973;3:48–51. 4715535 10.1007/BF02023852

[54] Rogers S, Keogh R, Pearson J. Hallucinations on demand: the utility of experimentally induced phenomena in hallucination research. Philos Trans Royal Soc B: Biol Sci. 2020;376:20200233. https://doi.org/10.1098/rstb.2020.0233 PMC7741072 33308076

[55] Cavanagh P. Using Illusions to Track the Emergence of Visual Perception. Annu Rev Vis Sci. 2024;10:1–22. 38871345 10.1146/annurev-vision-103023-012730

[56] Carter OL, Pettigrew JD, Burr DC, Alais D, Hasler F, Vollenweider FX. Psilocybin impairs high-level but not low-level motion perception:. Neuroreport. 2004;15:1947–1951. https://doi.org/10.1097/00001756-200408260-00023 15305143

[57] Castelo-Branco M, Mendes M, Silva F, et al Motion integration deficits are independent of magnocellular impairment in Parkinson’s disease. Neuropsychologia. 2009;47:314–320. https://doi.org/10.1016/j.neuropsychologia.2008.09.003 18822307

[58] Ezzati A, Khadjevand F, Zandvakili A, Abbassian A. Higher-level motion detection deficit in Parkinson’s disease. Brain Res. 2010;1320:143–151. https://doi.org/10.1016/j.brainres.2010.01.022 20083093

[59] Firbank MJ, Parikh J, Murphy N, et al Reduced occipital GABA in Parkinson disease with visual hallucinations. Neurology. 2018;91:e675–e685. https://doi.org/10.1212/WNL.0000000000006007 30021920 PMC6105043

[60] Sauer J, ffytche DH, Ballard C, Brown RG, Howard R. Differences between Alzheimer’s disease and dementia with Lewy bodies: an fMRI study of task-related brain activity. Brain. 2006;129:1780–1788. https://doi.org/10.1093/brain/awl102 16670180

[61] Stebbins GT, Goetz CG, Carrillo MC, et al Altered cortical visual processing in PD with hallucinations. Neurology. 2004;63:1409–1416. https://doi.org/10.1212/01.WNL.0000141853.27081.BD 15505157

[62] Taylor JP, Firbank MJ, He J, et al Visual cortex in dementia with Lewy bodies: magnetic resonance imaging study. Br J Psychiatry. 2012;200:491–498. https://doi.org/10.1192/bjp.bp.111.099432 22500014 PMC3365275

[63] Bowman AR, Bruce V, Colbourn CJ, Collerton D. Compensatory shifts in visual perception are associated with hallucinations in Lewy body disorders. Cognitive Res: Principles Implications. 2017;2:26. https://doi.org/10.1186/s41235-017-0063-6 PMC5442189 28603772

[64] Ffytche DH. Visual hallucinations and the charles bonnet syndrome. Curr Psychiatry Rep. 2005;7:168–179. https://doi.org/10.1007/s11920-005-0050-3 15935130

[65] Weil RS, Schrag AE, Warren JD, Crutch SJ, Lees AJ, Morris HR. Visual dysfunction in Parkinson’s disease. Brain. 2016;139:2827–2843. https://doi.org/10.1093/brain/aww175 27412389 PMC5091042

[66] Luke D, Terhune DB. The induction of synaesthesia with chemical agents: a systematic review. Front Psychol. 2013;4:753. https://doi.org/10.3389/fpsyg.2013.00753 24146659 PMC3797969

[67] Pagonabarraga J, Martinez-Horta S, Fernández de Bobadilla R, et al Minor hallucinations occur in drug-naive Parkinson’s disease patients, even from the premotor phase. Movement Disord. 2016;31:45–52. https://doi.org/10.1002/mds.26432 26408291

[68] Clegg BJ, Duncan GW, Khoo TK, et al Categorising visual hallucinations in early Parkinson’s disease. J Parkinson’s Dis. 2018;8:447–453. https://doi.org/10.3233/JPD-181338 30040741

[69] Llebaria G, Pagonabarraga J, Martínez-Corral M, et al Neuropsychological correlates of mild to severe hallucinations in Parkinson’s disease. Movement Disord. 2010;25:2785–2791. https://doi.org/10.1002/mds.23411 20960483

[70] Uchiyama M, Nishio Y, Yokoi K, Hosokai Y, Takeda A, Mori E. Pareidolia in Parkinson’s disease without dementia: a positron emission tomography study. Parkinsonism Relat Disord. 2015;21:603–609. https://doi.org/10.1016/j.parkreldis.2015.03.020 25864093

[71] Barnby JM, Park S, Baxter T, Rosen C, Brugger P, Alderson-Day B. The felt-presence experience: from cognition to the clinic. Lancet Psychiat. 2023;10:352–362. https://doi.org/10.1016/S2215-0366(23)00034-2 36990104

[72] Brederoo SG, Alderson-Day B, de Boer JN, Linszen MMJ, Sommer IEC. The experience of felt presence in a general population sample. Br J Psychiat. 2024;224:119–121. https://doi.org/10.1192/bjp.2024.7 PMC10933557 38470304

[73] Kamp KS, Steffen EM, Alderson-Day B, et al Sensory and quasi-sensory experiences of the deceased in bereavement: an interdisciplinary and integrative review. Schizophr Bull. 2020;46:1367–1381. https://doi.org/10.1093/schbul/sbaa113 33099644 PMC7707065

[74] Lifshitz M, Van Elk M, Luhrmann TM. Absorption and spiritual experience: a review of evidence and potential mechanisms. Conscious Cogn. 2019;73:102760. https://doi.org/10.1016/j.concog.2019.05.008 31228696

[75] Luhrmann TM, Morgain R. Prayer as inner sense cultivation: an attentional learning theory of spiritual experience. Ethos. 2012;40:359–389. https://doi.org/10.1111/j.1548-1352.2012.01266.x

[76] Wolff TJ, Ruffell S, Netzband N, Passie T. A phenomenology of subjectively relevant experiences induced by ayahuasca in Upper Amazon vegetalismo tourism. J. Psychedelic Stud. 2019;3:295–307. https://doi.org/10.1556/2054.2019.007

[77] Dupuis D. The socialization of hallucinations: cultural priors, social interactions, and contextual factors in the use of psychedelics. Transcult Psychiatry. 2022;59:625–637. https://doi.org/10.1177/13634615211036388 36367797 PMC9660275

[78] Dupuis D. Psychedelics as tools for belief transmission. Set, setting, suggestibility, and persuasion in the ritual use of hallucinogens. Front Psychol. 2021;12:730031. https://doi.org/10.3389/fpsyg.2021.730031 34887799 PMC8651242

[79] Barnes J, David A. Visual hallucinations in Parkinson’s disease: a review and phenomenological survey. J Neurol Neurosurg Psychiatry. 2001;70:727–733. https://doi.org/10.1136/jnnp.70.6.727 11385004 PMC1737396

[80] Onofrj M, Taylor JP, Monaco D, et al Visual hallucinations in PD and Lewy Body dementias: old and new hypotheses. Behav Neurol. 2013;27:479–493. https://doi.org/10.3233/BEN-129022 23242366 PMC5215646

[81] Mosimann UP, Rowan EN, Partington CE, et al Characteristics of visual hallucinations in Parkinson disease dementia and dementia with lewy bodies. Am J Geriatr Psychiatry. 2006;14:153–160. https://doi.org/10.1097/01.JGP.0000192480.89813.80 16473980

[82] Shanon B. Ayahuasca visualizations: a structural typology. J Consciousness Stud. 2002;9:3–30.

[83] Luke D. Discarnate entities and dimethyltryptamine (DMT): psychopharmacology, phenomenology and ontology. J Soc Psych Res. 2011;75:902.

[84] Shanon B. The epistemics of ayahuasca visions. Phenom Cogn Sci. 2010;9:263–280. https://doi.org/10.1007/s11097-010-9161-3

[85] Davis AK, Clifton JM, Weaver EG, Hurwitz ES, Johnson MW, Griffiths RR. Survey of entity encounter experiences occasioned by inhaled N,N-dimethyltryptamine: phenomenology, interpretation, and enduring effects. J Psychopharmacol. 2020;34:1008–1020. https://doi.org/10.1177/0269881120916143 32345112

[86] Lawrence DW, Carhart-Harris R, Griffiths R, Timmermann C. Phenomenology and content of the inhaled N, N-dimethyltryptamine (N, N-DMT) experience. Sci Rep. 2022;12:8562. https://doi.org/10.1038/s41598-022-11999-8 35610230 PMC9130218

[87] Lutkajtis A. Entity encounters and the therapeutic effect of the psychedelic mystical experience. J. Psychedelic Stud. 2021;4:171–178. https://doi.org/10.1556/2054.2020.00143

[88] Michael P, Luke D, Robinson O. An encounter with the self: a thematic and content analysis of the DMT experience from a naturalistic field study. Front Psychol. 2023;14:1083356. https://doi.org/10.3389/fpsyg.2023.1083356 37051610 PMC10083325

[89] Michael P, Luke D, Robinson O. An encounter with the other: a thematic and content analysis of DMT experiences from a naturalistic field study. Front Psychol. 2021;12:720717. https://doi.org/10.3389/fpsyg.2021.720717 34975614 PMC8716686

[90] Suzuki K, Seth AK, Schwartzman DJ. Modelling phenomenological differences in aetiologically distinct visual hallucinations using deep neural networks. Front Hum Neurosci. 2024;17:1159821. https://doi.org/10.3389/fnhum.2023.1159821 38234594 PMC10791985

[91] Waters F, Chiu V, Atkinson A, Blom JD. Severe sleep deprivation causes hallucinations and a gradual progression toward psychosis with increasing time awake. Front Psychiatry. 2018;9:303. https://doi.org/10.3389/fpsyt.2018.00303 30042701 PMC6048360

[92] Bowman AR, Bruce V, Colbourn CJ, Collerton D. Compensatory shifts in visual perception are associated with hallucinations in Lewy body disorders. Cognitive Res: Principles Implicat. 2017;2:1–9. https://doi.org/10.1186/s41235-017-0063-6 PMC5442189 28603772

[93] Vollenweider FX, Preller KH. Psychedelic drugs: neurobiology and potential for treatment of psychiatric disorders. Nat Rev Neurosci. 2020;21:611–624. https://doi.org/10.1038/s41583-020-0367-2 32929261

[94] Hermle L, Fünfgeld M, Oepen G, et al Mescaline-induced psychopathological, neuropsychological, and neurometabolic effects in normal subjects: Experimental psychosis as a tool for psychiatric research. Biol Psychiatry. 1992;32:976–991. https://doi.org/10.1016/0006-3223(92)90059-9 1467389

[95] Gouzoulis-Mayfrank E, Schreckenberger M, Sabri O, et al Neurometabolic effects of psilocybin, 3,4-methylenedioxyethylamphetamine (MDE) and d-methamphetamine in healthy volunteers a double-blind, placebo-controlled PET study with [18F]FDG. Neuropsychopharmacology.. 1999;20:565–581. https://doi.org/10.1016/S0893-133X(98)00089-X 10327426

[96] Vollenweider FX, Leenders KL, Scharfetter C, Maguire P, Stadelmann O, Angst J. Positron emission tomography and fluorodeoxyglucose studies of metabolic hyperfrontality and psychopathology in the psilocybin model of psychosis. Neuropsychopharmacology. 1997;16:357–372. https://doi.org/10.1016/S0893-133X(96)00246-1 9109107

[97] Lewis CR, Preller KH, Kraehenmann R, Michels L, Staempfli P, Vollenweider FX. Two dose investigation of the 5-HT-agonist psilocybin on relative and global cerebral blood flow. Neuroimage. 2017;159:70–78. https://doi.org/10.1016/j.neuroimage.2017.07.020 28711736

[98] Preller KH, Burt JB, Ji JL, et al Changes in global and thalamic brain connectivity in LSD-induced altered states of consciousness are attributable to the 5-HT2A receptor. Hunt LT, Behrens TE, eds. eLife. 2018;7:e35082. https://doi.org/10.7554/eLife.35082 30355445 PMC6202055

[99] Cioli C, Abdi H, Beaton D, Burnod Y, Mesmoudi S. Differences in human cortical gene expression match the temporal properties of large-scale functional networks. PLoS One. 2014;9:e115913. https://doi.org/10.1371/journal.pone.0115913 25546015 PMC4278769

[100] Golland Y, Golland P, Bentin S, Malach R. Data-driven clustering reveals a fundamental subdivision of the human cortex into two global systems. Neuropsychologia. 2008;46:540–553. https://doi.org/10.1016/j.neuropsychologia.2007.10.003 18037453 PMC4468071

[101] Nagano-Saito A, Washimi Y, Arahata Y, et al Visual hallucination in Parkinson's disease with FDG PET. Mov Disord. 2004;19:801–806. https://doi.org/10.1002/mds.20086 15254938

[102] Boecker H, Ceballos-Baumann AO, Bartenstein P, et al Metabolic alterations in patients with Parkinson disease and visual hallucinations. Arch Neurol. 2007;64:984–988. https://doi.org/10.1001/archneur.64.7.984 17620488

[103] Firbank MJ, Lloyd J, O’Brien JT. The relationship between hallucinations and FDG-PET in dementia with Lewy bodies. Brain Imaging Behav. 2016;10:636–639. https://doi.org/10.1007/s11682-015-9429-4 26239998

[104] Imamura T, Ishii K, Hirono N, et al Visual hallucinations and regional cerebral metabolism in dementia with Lewy bodies (DLB). Neuroreport. 1999;10:1903–1907. https://doi.org/10.1097/00001756-199906030-00007 10501530

[105] Perneczky R, Drzezga A, Boecker H, Förstl H, Kurz A, Häussermann P. Cerebral metabolic dysfunction in patients with dementia with Lewy bodies and visual hallucinations. Dement Geriatr Cogn Disord. 2008;25:531–538. https://doi.org/10.1159/000132271 18477846

[106] Sato T, Nishio Y, Suzuki M, et al Deep gray matter hyperperfusion with occipital hypoperfusion in dementia with Lewy bodies. Eur J Neurol. 2007;14:1299–1301. https://doi.org/10.1111/j.1468-1331.2007.01976.x 17877736

[107] Vignando M, Ffytche D, Lewis SJG, et al Mapping brain structural differences and neuroreceptor correlates in Parkinson’s disease visual hallucinations. Nat Commun. 2022;13:519. https://doi.org/10.1038/s41467-022-28087-0 35082285 PMC8791961

[108] Thomas GEC, Zeidman P, Sultana T, Zarkali A, Razi A, Weil RS. Changes in both top-down and bottom-up effective connectivity drive visual hallucinations in Parkinson’s disease. Brain Commun. 2023;5:fcac329. https://doi.org/10.1093/braincomms/fcac329 36601626 PMC9798302

[109] Stoliker D, Preller KH, Novelli L, et al Neural mechanisms of psychedelic visual imagery. Mol Psychiatry. 2024;30:1259–1266. https://doi.org/10.1038/s41380-024-02632-3 38862674 PMC11919690

[110] Tipado Z, Razi A, Heitmann S, et al Visual hallucinations originating in the retinofugal pathway under clinical and psychedelic conditions. Eur Neuropsychopharmacol. 2024;85:10–20. https://doi.org/10.1016/j.euroneuro.2023.12.005 38648694

[111] Corlett PR, Frith CD, Fletcher PC. From drugs to deprivation: a Bayesian framework for understanding models of psychosis. Psychopharmacology (Berl). 2009;206:515–530. https://doi.org/10.1007/s00213-009-1561-0 19475401 PMC2755113

[112] Lindahl JR, Kaplan CT, Winget EM, Britton WB. A phenomenology of meditation-induced light experiences: traditional Buddhist and neurobiological perspectives. Front Psychol. 2014;4:973. https://doi.org/10.3389/fpsyg.2013.00973 24427148 PMC3879457

[113] Collerton D, Barnes J, Diederich NJ, et al Understanding visual hallucinations: a new synthesis. Neurosci Biobehav Rev. 2023;150:105208. https://doi.org/10.1016/j.neubiorev.2023.105208 37141962

[114] Diederich NJ, Goetz CG, Stebbins GT. Repeated visual hallucinations in Parkinson’s disease as disturbed external/internal perceptions: focused review and a new integrative model. Movement Disord. 2005;20:130–140. https://doi.org/10.1002/mds.20308 15486924

[115] Shine JM, Halliday GM, Naismith SL, Lewis SJG. Visual misperceptions and hallucinations in Parkinson’s disease: dysfunction of attentional control networks? Movement Disord.. 2011;26:2154–2159. https://doi.org/10.1002/mds.23896 21953814

[116] Shine JM, O’Callaghan C, Halliday GM, Lewis SJG. Tricks of the mind: visual hallucinations as disorders of attention. Prog Neurobiol. 2014;116:58–65. https://doi.org/10.1016/j.pneurobio.2014.01.004 24525149

[117] Friston KJ. Hallucinations and perceptual inference. Behav Brain Sci. 2005;28:764–766. https://doi.org/10.1017/S0140525X05290131

[118] Onofrj M, Espay AJ, Bonanni L, Delli Pizzi S, Sensi SL. Hallucinations, somatic-functional disorders of PD-DLB as expressions of thalamic dysfunction. Movement Disord. 2019;34:1100–1111. https://doi.org/10.1002/mds.27781 31307115 PMC6707070

[119] Barnes J, Boubert L, Harris J, Lee A, David AS. Reality monitoring and visual hallucinations in Parkinson’s disease. Neuropsychologia. 2003;41:565–574. https://doi.org/10.1016/s0028-3932(02)00182-3 12559149

[120] Ffytche DH. The hodology of hallucinations. Cortex. 2008;44:1067–1083. https://doi.org/10.1016/j.cortex.2008.04.005 18586234

[121] Burke W. The neural basis of Charles Bonnet hallucinations: a hypothesis. J Neurol, Neurosurg Psychiat. 2002;73:535–541. https://doi.org/10.1136/jnnp.73.5.535 12397147 PMC1738134

[122] Doss MK, Madden MB, Gaddis A, et al Models of psychedelic drug action: modulation of cortical-subcortical circuits. Brain. 2022;145:441–456. https://doi.org/10.1093/brain/awab406 34897383 PMC9014750

[123] McCulloch DEW, Knudsen GM, Barrett FS, et al Psychedelic resting-state neuroimaging: a review and perspective on balancing replication and novel analyses. Neurosci Biobehav Rev. 2022;138:104689. https://doi.org/10.1016/j.neubiorev.2022.104689 35588933

[124] Shine JM, Muller AJ, O’Callaghan C, Hornberger M, Halliday GM, Lewis SJ. Abnormal connectivity between the default mode and the visual system underlies the manifestation of visual hallucinations in Parkinson’s disease: a task-based fMRI study. npj Parkinson's Dis. 2015;1:1–8. https://doi.org/10.1038/npjparkd.2015.3 PMC5516559 28725679

[125] Siegel JS, Subramanian S, Perry D, et al Psilocybin desynchronizes the human brain. Nature. 2024;632:131–138. https://doi.org/10.1038/s41586-024-07624-5 39020167 PMC11291293

[126] Dujardin K, Roman D, Baille G, et al What can we learn from fMRI capture of visual hallucinations in Parkinson’s disease? Brain Imaging Behavior. 2020;14:329–335. https://doi.org/10.1007/s11682-019-00185-6 31444780

[127] Pezzoli S, Sánchez-Valle R, Solanes A, et al Neuroanatomical and cognitive correlates of visual hallucinations in Parkinson’s disease and dementia with Lewy bodies: voxel-based morphometry and neuropsychological meta-analysis. Neurosci Biobehav Rev. 2021;128:367–382. https://doi.org/10.1016/j.neubiorev.2021.06.030 34171324

[128] Franciotti R, Delli Pizzi S, Perfetti B, et al Default mode network links to visual hallucinations: a comparison between Parkinson’s disease and multiple system atrophy. Movement Disord. 2015;30:1237–1247. https://doi.org/10.1002/mds.26285 26094856

[129] Yao N, Shek-Kwan Chang R, Cheung C, et al The default mode network is disrupted in parkinson’s disease with visual hallucinations. Hum Brain Mapp. 2014;35:5658–5666. https://doi.org/10.1002/hbm.22577 24985056 PMC4657500

[130] Shinozuka K, Jerotic K, Mediano P, et al Synergistic, multi-level understanding of psychedelics: three systematic reviews and meta-analyses of their pharmacology, neuroimaging and phenomenology. Transl Psychiatry. 2024;14:1–16. https://doi.org/10.1038/s41398-024-03187-1 39632810 PMC11618481

[131] Carhart-Harris RL, Friston KJ. REBUS and the Anarchic brain: toward a unified model of the brain action of psychedelics. Pharmacol Rev. 2019;71:316–344. https://doi.org/10.1124/pr.118.017160 31221820 PMC6588209

[132] Barrett FS, Carbonaro TM, Hurwitz E, Johnson MW, Griffiths RR. Double-blind comparison of the two hallucinogens psilocybin and dextromethorphan: effects on cognition. Psychopharmacology (Berl). 2018;235:2915–2927. https://doi.org/10.1007/s00213-018-4981-x 30062577 PMC6162157

[133] Pokorny T, Duerler P, Seifritz E, Vollenweider FX, Preller KH. LSD acutely impairs working memory, executive functions, and cognitive flexibility, but not risk-based decision-making. Psychol Med. 2020;50:2255–2264. https://doi.org/10.1017/S0033291719002393 31500679

[134] Puntambekar I, Foley J A. Neuropsychological correlates of visual hallucinatory phenomena in Lewy body disease. Int J Geriatr Psychiatry. 2023;38:e5950. https://doi.org/10.1002/gps.5950 37334515

[135] Heller NH, Barrett FS. Teaching a new dog old tricks: bringing rigor, grounding, and specificity to psychedelic neuropsychopharmacology. Neuropsychopharmacology. 2024;50:324–325. https://doi.org/10.1038/s41386-024-01954-8 39107542 PMC11526001

[136] Nieto-Escamez F, Obrero-Gaitán E, Cortés-Pérez I. Visual dysfunction in Parkinson’s disease. Brain Sciences. 2023;13:1173. https://doi.org/10.3390/brainsci13081173 37626529 PMC10452537

[137] Ekker MS, Janssen S, Seppi K, et al Ocular and visual disorders in Parkinson’s disease: common but frequently overlooked. Parkinsonism Relat Disord. 2017;40:1–10. https://doi.org/10.1016/j.parkreldis.2017.02.014 28284903

[138] Urwyler P, Nef T, Killen A, et al Visual complaints and visual hallucinations in Parkinson’s disease. Parkinsonism Relat Disord. 2014;20:318–322. https://doi.org/10.1016/j.parkreldis.2013.12.009 24405755

[139] Holroyd S, Wooten GF. Preliminary fMRI evidence of visual system dysfunction in parkinson’s disease patients with visual hallucinations. J Neuropsychiatry Clin Neurosci. 2006;18:402–404. https://doi.org/10.1176/jnp.2006.18.3.402 16963591

[140] Meppelink AM, de Jong BM, Renken R, Leenders KL, Cornelissen FW, van Laar T. Impaired visual processing preceding image recognition in Parkinson’s disease patients with visual hallucinations. Brain. 2009;132:2980–2993. https://doi.org/10.1093/brain/awp223 19755518

[141] Vignando M, ffytche D, Mazibuko N, et al Visual mismatch negativity in Parkinson’s psychosis and potential for testing treatment mechanisms. Brain Commun. 2024;6:fcae291. https://doi.org/10.1093/braincomms/fcae291 39355002 PMC11443450

[142] Kometer M, Cahn BR, Andel D, Carter OL, Vollenweider FX. The 5-HT2A/1A agonist psilocybin disrupts modal object completion associated with visual hallucinations. Biol Psychiatry. 2011;69:399–406. https://doi.org/10.1016/j.biopsych.2010.10.002 21126732

[143] Kometer M, Schmidt A, Jäncke L, Vollenweider FX. Activation of Serotonin 2A Receptors underlies the psilocybin-induced effects on α oscillations, N170 visual-evoked potentials, and visual hallucinations. J Neurosci. 2013;33:10544–10551. https://doi.org/10.1523/JNEUROSCI.3007-12.2013 23785166 PMC6618596

[144] Michaiel AM, Parker PRL, Niell CM. A Hallucinogenic serotonin-2A receptor agonist reduces visual response gain and alters temporal dynamics in mouse V1. Cell Reports. 2019;26:3475–3483.e4. https://doi.org/10.1016/j.celrep.2019.02.104 30917304 PMC6559379

[145] Bernasconi F, Schmidt A, Pokorny T, Kometer M, Seifritz E, Vollenweider FX. Spatiotemporal brain dynamics of emotional face processing modulations induced by the serotonin 1A/2A receptor agonist psilocybin. Cerebral Cortex (New York, N.Y. : 1991). 2014;24:3221–3231. https://doi.org/10.1093/cercor/bht178 23861318

[146] Schmidt A, Kometer M, Bachmann R, Seifritz E, Vollenweider F. The NMDA antagonist ketamine and the 5-HT agonist psilocybin produce dissociable effects on structural encoding of emotional face expressions. Psychopharmacology (Berl). 2013;225:227–239. https://doi.org/10.1007/s00213-012-2811-0 22836372

[147] Rossion B, Joyce CA, Cottrell GW, Tarr MJ. Early lateralization and orientation tuning for face, word, and object processing in the visual cortex. Neuroimage. 2003;20:1609–1624. https://doi.org/10.1016/j.neuroimage.2003.07.010 14642472

[148] Kraehenmann R, Schmidt A, Friston K, Preller KH, Seifritz E, Vollenweider FX. The mixed serotonin receptor agonist psilocybin reduces threat-induced modulation of amygdala connectivity. Neuroimage Clin. 2016;11:53–60. https://doi.org/10.1016/j.nicl.2015.08.009 26909323 PMC4732191

[149] Jardri R, Pouchet A, Pins D, Thomas P. Cortical activations during auditory verbal hallucinations in schizophrenia: a coordinate-based meta-analysis. Am J Psychiatry. 2011;168:73–81. https://doi.org/10.1176/appi.ajp.2010.09101522 20952459

[150] Ffytche DH, Howard RJ, Brammer MJ, David A, Woodruff P, Williams S. The anatomy of conscious vision: an fMRI study of visual hallucinations. Nat Neurosci. 1998;1:738–742. https://doi.org/10.1038/3738 10196592

[151] de Pierrefeu A, Fovet T, Hadj-Selem F, et al Prediction of activation patterns preceding hallucinations in patients with schizophrenia using machine learning with structured sparsity. Hum Brain Mapp. 2018;39:1777–1788. https://doi.org/10.1002/hbm.23953 29341341 PMC6866438

[152] Hugdahl K, Craven AR, Johnsen E, et al Neural activation in the ventromedial prefrontal cortex precedes conscious experience of being in or out of a transient hallucinatory state. Schizophr Bull. 2023;49:S58–S67. https://doi.org/10.1093/schbul/sbac028 35596662 PMC9960028

[153] Roseman L, Sereno MI, Leech R, et al LSD alters eyes-closed functional connectivity within the early visual cortex in a retinotopic fashion. Hum Brain Mapp. 2016;37:3031–3040. https://doi.org/10.1002/hbm.23224 27125770 PMC6867480

[154] Chweitzer A, Geblewicz E, Liberson W. V. Étude de l’électrencéphalogramme humain dans un cas d’intoxication mescalinique. Published online 1936. https://doi.org/10.3406/psy.1936.5381

[155] Endo K. Experimental study of mescalin intoxication on relation between clinical picture and e.g. in man. Folia Psychiatrica et Neurologica Japonica. 1952;6:104–113. https://doi.org/10.1111/j.1440-1819.1952.tb01687.x 13060417

[156] Shirahashi K. Electroencephalographic study of mental disturbances experimentally induced by Lsd25. Psychiatry Clin Neurosci. 1960;14:140–155. https://doi.org/10.1111/j.1440-1819.1960.tb02237.x

[157] Brown BB. Effect of LSD on visually evoked responses to color in visualizer and non-visualizer subjects. Electroencephalogr Clin Neurophysiol. 1969;27:356–363. https://doi.org/10.1016/0013-4694(69)91444-8 4186733

[158] Akpinar S, Itil T, Marasa J, Marrazzi A. Blocking effect of chlorpromazine on LSD-25 induced clinical and digital computer analyzed EEG changes. Clin Electroencephalogr. 1972;3:224–232. https://doi.org/10.1177/155005947200300403

[159] Wikler A. A pharmacologic analysis of the functions of the spontaneous electrical activity of the cerebral cortex. J Nerv Ment Dis. 1954;120:157–175. 13233958

[160] Bercel NA, Travis LE, Olinger LB, Dreikurs E, Polos MG. Model psychoses induced by LSD-25 in normals: i. psychophysiological investigations, with special reference to the mechanism of the paranoid reaction. AMA Archives of Neurology & Psychiatry. 1956;75:588–611. https://doi.org/10.1001/archneurpsyc.1956.02330240026003 13325989

[161] Gastaut H, Ferrer S, Castells C, Lesèvre N, Luschnat K. Action de la diéthylamide de l’acide d-lysergique (LSD 25) sur les fonctions psychiques et I’électroencéphalogramme. Stereotact Funct Neurosurg. 1953;13:102–120. https://doi.org/10.1159/000105400 13060015

[162] Elkes J, Elkes C, Bradley PB. The effect of some drugs on the electrical activity of the brain, and on behaviour. The Journal of mental science. 1954;100:125–128. https://doi.org/10.1192/bjp.100.418.125 13152527

[163] Taylor JP, Firbank M, Barnett N, et al Visual hallucinations in dementia with Lewy bodies: transcranial magnetic stimulation study. Br J Psychiat J Mental Sci. 2011;199:492–500. https://doi.org/10.1192/bjp.bp.110.090373 PMC3227808 22016436

[164] Taylor JP, Firbank M, O’Brien JT. Visual cortical excitability in dementia with Lewy bodies. Br J Psychiatry. 2016;208:497–498. https://doi.org/10.1192/bjp.bp.114.152736 26541688 PMC4853644

[165] Khundakar AA, Hanson PS, Erskine D, et al Analysis of primary visual cortex in dementia with Lewy bodies indicates GABAergic involvement associated with recurrent complex visual hallucinations. Acta Neuropathol Commun. 2016;4:66. https://doi.org/10.1186/s40478-016-0334-3 27357212 PMC4928325

[166] Lunghi C, Emir UE, Morrone MC, Bridge H. Short-term monocular deprivation alters GABA in the adult human visual cortex. Curr Biol. 2015;25:1496–1501. https://doi.org/10.1016/j.cub.2015.04.021 26004760 PMC5040500

[167] Haegens S, Barczak A, Musacchia G, et al Laminar profile and physiology of the α rhythm in primary visual, auditory, and somatosensory regions of neocortex. J Neurosci. 2015;35:14341–14352. https://doi.org/10.1523/JNEUROSCI.0600-15.2015 26490871 PMC4683691

[168] Samaha J, Iemi L, Haegens S, Busch NA. Spontaneous brain oscillations and perceptual decision-making. Trends Cogn Sci. 2020;24:639–653. https://doi.org/10.1016/j.tics.2020.05.004 32513573

[169] Carhart-Harris RL, Muthukumaraswamy S, Roseman L, et al Neural correlates of the LSD experience revealed by multimodal neuroimaging. Proc Natl Acad Sci USA. 2016;113:4853–4858. https://doi.org/10.1073/pnas.1518377113 27071089 PMC4855588

[170] Muthukumaraswamy SD, Carhart-Harris RL, Moran RJ, et al Broadband cortical desynchronization underlies the human psychedelic state. J Neurosci. 2013;33:15171–15183. https://doi.org/10.1523/JNEUROSCI.2063-13.2013 24048847 PMC6618409

[171] Riba J, Anderer P, Morte A, et al Topographic pharmaco-EEG mapping of the effects of the South American psychoactive beverage ayahuasca in healthy volunteers. Br J Clin Pharmacol. 2002;53:613–628. https://doi.org/10.1046/j.1365-2125.2002.01609.x 12047486 PMC1874340

[172] Riba J, Anderer P, Jané F, Saletu B, Barbanoj MJ. Effects of the South American psychoactive beverage ayahuasca on regional brain electrical activity in humans: a functional neuroimaging study using low-resolution electromagnetic tomography. Neuropsychobiology. 2004;50:89–101. https://doi.org/10.1159/000077946 15179026

[173] Schenberg EE, Alexandre JFM, Filev R, et al Acute biphasic effects of ayahuasca. PLoS One. 2015;10:e0137202. https://doi.org/10.1371/journal.pone.0137202 26421727 PMC4589238

[174] Stuckey DE, Lawson R, Luna LE. EEG gamma coherence and other correlates of subjective reports during ayahuasca experiences. J Psychoactive Drugs. 2005;37:163–178. https://doi.org/10.1080/02791072.2005.10399798 16149330

[175] Timmermann C, Roseman L, Schartner M, et al Neural correlates of the DMT experience assessed with multivariate EEG. Sci Rep. 2019;9:16324. https://doi.org/10.1038/s41598-019-51974-4 31745107 PMC6864083

[176] Timmermann C, Roseman L, Haridas S, et al Human brain effects of DMT assessed via EEG-fMRI. Proc Natl Acad Sci USA. 2023;120:e2218949120. https://doi.org/10.1073/pnas.2218949120 36940333 PMC10068756

[177] Muthukumaraswamy SD, Liley DTJ. 1/f electrophysiological spectra in resting and drug-induced states can be explained by the dynamics of multiple oscillatory relaxation processes. Neuroimage. 2018;179:582–595. https://doi.org/10.1016/j.neuroimage.2018.06.068 29959047

[178] Gao R, Peterson EJ, Voytek B. Inferring synaptic excitation/inhibition balance from field potentials. Neuroimage. 2017;158:70–78. https://doi.org/10.1016/j.neuroimage.2017.06.078 28676297

[179] Di Russo F, Martínez A, Hillyard SA. Source analysis of event-related cortical activity during visuo-spatial Attention. Cerebral cortex (New York, N.Y. : 1991). 2003;13:486–499. https://doi.org/10.1093/cercor/13.5.486 12679295

[180] Azimi Z, Barzan R, Spoida K, et al Separable gain control of ongoing and evoked activity in the visual cortex by serotonergic input. Lottem E, Gold JI, Lorincz M, eds. eLife. 2020;9:e53552. https://doi.org/10.7554/eLife.53552 32252889 PMC7138610

[181] Seillier L, Lorenz C, Kawaguchi K, et al Serotonin decreases the gain of visual responses in awake macaque V1. J Neurosci. 2017;37:11390–11405. https://doi.org/10.1523/JNEUROSCI.1339-17.2017 29042433 PMC5700422

[182] Chen L, Li X, Tjia M, Thapliyal S. Homeostatic plasticity and excitation-inhibition balance: the good, the bad, and the ugly. Curr Opin Neurobiol. 2022;75:102553. https://doi.org/10.1016/j.conb.2022.102553 35594578 PMC9477500

[183] Marschall TM, Brederoo SG, Ćurčić-Blake B, Sommer IEC. Deafferentation as a cause of hallucinations. Curr Opin Psychiatry. 2020;33:206–211. https://doi.org/10.1097/YCO.0000000000000586 32040043

[184] Wen W, Turrigiano GG. Keeping your brain in balance: homeostatic regulation of network function. Annu Rev Neurosci. 2024;47:41–61. https://doi.org/10.1146/annurev-neuro-092523-110001 38382543

[185] Hensch TK. Critical period plasticity in local cortical circuits. Nat Rev Neurosci. 2005;6:877–888. https://doi.org/10.1038/nrn1787 16261181

[186] Lepow L, Morishita H, Yehuda R. Critical period plasticity as a framework for psychedelic-assisted psychotherapy. Front Neurosci. 2021;15:710004. https://doi.org/10.3389/fnins.2021.710004 34616272 PMC8488335

[187] Nardou R, Sawyer E, Song YJ, et al Psychedelics reopen the social reward learning critical period. Nature. 2023;618:790–798. https://doi.org/10.1038/s41586-023-06204-3 37316665 PMC10284704

[188] Moliner R, Girych M, Brunello CA, et al Psychedelics promote plasticity by directly binding to BDNF receptor TrkB. Nat Neurosci. 2023;26:1032–1041. https://doi.org/10.1038/s41593-023-01316-5 37280397 PMC10244169

[189] Scarlatti F. The relationship between plasticity in the primary visual cortex and the hallucinatory persisting perception disorder. PsyArXiv. 2023.

[190] Klein JC, Eggers C, Kalbe E, et al Neurotransmitter changes in dementia with Lewy bodies and Parkinson disease dementia in vivo. Neurology. 2010;74:885–892. https://doi.org/10.1212/WNL.0b013e3181d55f61 20181924

[191] Walker Z, Costa DC, Walker RWH, et al Striatal dopamine transporter in dementia with Lewy bodies and Parkinson disease. Neurology. 2004;62:1568–1572. https://doi.org/10.1212/01.WNL.0000123248.39847.1D 15136683

[192] Fedorova TD, Knudsen K, Horsager J, et al Dopaminergic dysfunction is more symmetric in dementia with lewy bodies compared to Parkinson’s disease. Journal of Parkinson’s Disease. 2023;13:515–523. https://doi.org/10.3233/JPD-230001 PMC10357144 37212074

[193] Janzen J, van ‘t Ent D, Lemstra AW, Berendse HW, Barkhof F, Foncke EMJ. The pedunculopontine nucleus is related to visual hallucinations in Parkinson’s disease: preliminary results of a voxel-based morphometry study. J Neurol. 2012;259:147–154. https://doi.org/10.1007/s00415-011-6149-z 21717194 PMC3251778

[194] Barrett MJ, Blair JC, Sperling SA, Smolkin ME, Druzgal TJ. Baseline symptoms and basal forebrain volume predict future psychosis in early Parkinson disease. Neurology. 2018;90:e1618–e1626. https://doi.org/10.1212/WNL.0000000000005421 29618627

[195] Celesia GG, Barr AN. Psychosis and other psychiatric manifestations of levodopa therapy. Arch Neurol. 1970;23:193–200. https://doi.org/10.1001/archneur.1970.00480270003001 5456717

[196] Sharf B, Moskovitz C, Lupton MD, Klawans HL. Dream phenomena induced by chronic levodopa therapy. J Neural Transm. 1978;43:143–151. https://doi.org/10.1007/BF01579073 104005

[197] Moskovitz C, Moses H, Klawans HL. Levodopa-induced psychosis: a kindling phenomenon. Am J Psychiatry. 1978;135:669–675. https://doi.org/10.1176/ajp.135.6.669 655276

[198] Taylor JP, Collerton D, LeBeau F, Perry E. Cholinergic pathology in dementia with Lewy bodies. In: Kosaka K, ed. Dementia with Lewy Bodies: Clinical and Biological Aspects. Springer, Japan; 2017:23–39. https://doi.org/10.1007/978-4-431-55948-1_3

[199] Perry EK, Marshall E, Kerwin J, et al Evidence of a monoaminergic-cholinergic imbalance related to visual hallucinations in Lewy body dementia. J Neurochem. 1990;55:1454–1456. https://doi.org/10.1111/j.1471-4159.1990.tb03162.x 1697897

[200] Marshall L, Mathys C, Ruge D, et al Pharmacological fingerprints of contextual uncertainty. PLoS Biol. 2016;14:e1002575. https://doi.org/10.1371/journal.pbio.1002575 27846219 PMC5113004

[201] Moran RJ, Campo P, Symmonds M, Stephan KE, Dolan RJ, Friston KJ. Free energy, precision and learning: the role of cholinergic neuromodulation. J Neurosci. 2013;33:8227–8236. https://doi.org/10.1523/JNEUROSCI.4255-12.2013 23658161 PMC4235126

[202] Yu AJ, Dayan P. Uncertainty, neuromodulation, and attention. Neuron. 2005;46:681–692. https://doi.org/10.1016/j.neuron.2005.04.026 15944135

[203] Corlett PR, Horga G, Fletcher PC, Alderson-Day B, Schmack K, Powers AR. Hallucinations and strong priors. Trends Cogn Sci. 2019;23:114–127. https://doi.org/10.1016/j.tics.2018.12.001 30583945 PMC6368358

[204] Schmack K, Bosc M, Ott T, Sturgill JF, Kepecs A. Striatal dopamine mediates hallucination-like perception in mice. Science. 2021;372:eabf4740. https://doi.org/10.1126/science.abf4740 33795430

[205] Perry EK, Perry RH. Acetylcholine and hallucinations - disease-related compared to drug-induced alterations in human consciousness. Brain Cogn. 1995;28:240–258. https://doi.org/10.1006/brcg.1995.1255 8546852

[206] Warburton DM, Wesnes K, Edwards J, Larrad D. Scopolamine and the sensory conditioning of hallucinations. Neuropsychobiology. 2009;14:198–202. https://doi.org/10.1159/000118227 3835496

[207] Dykstra LA, Appel JB. Effects of LSD on auditory perception: a signal detection analysis. Psychopharmacologia. 1974;34:289–307. https://doi.org/10.1007/BF00422553 4812655

[208] Hawkins T, Berman BD. Pimavanserin. Neurol Clin Pract. 2017;7:157–162. https://doi.org/10.1212/CPJ.0000000000000342 29185542 PMC5669409

[209] McFarland K, Price DL, Bonhaus DW. Pimavanserin, a 5-HT2A inverse agonist, reverses psychosis-like behaviors in a rodent model of Parkinson’s disease. Behav Pharmacol. 2011;22:681–692. https://doi.org/10.1097/FBP.0b013e32834aff98 21921840

[210] Buchborn T, Lyons T, Song C, Feilding A, Knöpfel T. Cortical correlates of psychedelic-induced shaking behavior revealed by voltage imaging. Int J Mol Sci. 2023;24:9463. https://doi.org/10.3390/ijms24119463 37298417 PMC10253917

[211] Halberstadt AL, Chatha M, Klein AK, Wallach J, Brandt SD. Correlation between the potency of hallucinogens in the mouse head-twitch response assay and their behavioral and subjective effects in other species. Neuropharmacology. 2020;167:107933. https://doi.org/10.1016/j.neuropharm.2019.107933 31917152 PMC9191653

[212] Huot P, Fox SH. The serotonergic system in motor and non-motor manifestations of Parkinson’s disease. Exp Brain Res. 2013;230:463–476. https://doi.org/10.1007/s00221-013-3621-2 23811734

[213] Huot P, Sgambato-Faure V, Fox SH, McCreary AC. Serotonergic approaches in Parkinson’s disease: translational perspectives, an update. ACS Chem Neurosci. 2017;8:973–986. https://doi.org/10.1021/acschemneuro.6b00440 28460160

[214] Huot P, Johnston TH, Darr T, et al Increased 5-HT2A receptors in the temporal cortex of parkinsonian patients with visual hallucinations. Movement Disord.. 2010;25:1399–1408. https://doi.org/10.1002/mds.23083 20629135

[215] Ballanger B, Strafella AP, van Eimeren T, et al Serotonin 2A receptors and visual hallucinations in Parkinson disease. Arch Neurol. 2010;67:416–421. https://doi.org/10.1001/archneurol.2010.35 20385906

[216] Basura GJ, Walker PD. Serotonin 2A receptor mRNA levels in the neonatal dopamine-depleted rat striatum remain upregulated following suppression of serotonin hyperinnervation. Brain Res Dev Brain Res. 1999;116:111–117. https://doi.org/10.1016/s0165-3806(99)00066-8 10446352

[217] Laprade N, Radja F, Reader TA, Soghomonian JJ. Dopamine receptor agonists regulate levels of the serotonin 5-HT2A receptor and its mRNA in a subpopulation of rat striatal neurons. J Neurosci. 1996;16:3727–3736. https://doi.org/10.1523/JNEUROSCI.16-11-03727.1996 8642415 PMC6578831

[218] Numan S, Lundgren KH, Wright DE, Herman JP, Seroogy KB. Increased expression of 5HT2 receptor mRNA in rat striatum following 6-OHDA lesions of the adult nigrostriatal pathway. Brain Res Mol Brain Res. 1995;29:391–396. https://doi.org/10.1016/0169-328x(95)00004-c 7609629

[219] Radja F, Descarries L, Dewar KM, Reader TA. Serotonin 5-HT1 and 5-HT2 receptors in adult rat brain after neonatal destruction of nigrostriatal dopamine neurons: a quantitative autoradiographic study. Brain Res. 1993;606:273–285. https://doi.org/10.1016/0006-8993(93)90995-y 8490720

[220] Zhang X, Andren PE, Svenningsson P. Changes on 5-HT2 receptor mRNAs in striatum and subthalamic nucleus in Parkinson’s disease model. Physiol Behav. 2007;92:29–33. https://doi.org/10.1016/j.physbeh.2007.05.033 17588622

[221] Li L, Qiu G, Ding S, Zhou FM. Serotonin hyperinnervation and upregulated 5-HT2A receptor expression and motor-stimulating function in nigrostriatal dopamine-deficient Pitx3 mutant mice. Brain Res. 2013;1491:236–250. https://doi.org/10.1016/j.brainres.2012.11.010 23159831 PMC3596263

[222] Wang S, Zhang QJ, Liu J, et al In vivo effects of activation and blockade of 5-HT(2A/2C) receptors in the firing activity of pyramidal neurons of medial prefrontal cortex in a rodent model of Parkinson’s disease. Exp Neurol. 2009;219:239–248. https://doi.org/10.1016/j.expneurol.2009.05.029 19500571

[223] Zhang QJ, Wang S, Liu J, et al Unilateral lesion of the nigrostriatal pathway decreases the response of interneurons in medial prefrontal cortex to 5-HT 2A/2C receptor stimulation in the rat. Brain Res. 2010;1312:127–137. https://doi.org/10.1016/j.brainres.2009.11.052 19948151

[224] Aghajanian GK, Marek GJ. Serotonin induces excitatory postsynaptic potentials in apical dendrites of neocortical pyramidal cells. Neuropharmacology. 1997;36:589–599. https://doi.org/10.1016/s0028-3908(97)00051-8 9225284

[225] Aghajanian GK, Marek GJ. Serotonin, via 5-HT2A receptors, increases EPSCs in layer V pyramidal cells of prefrontal cortex by an asynchronous mode of glutamate release. Brain Res. 1999;825:161–171. https://doi.org/10.1016/s0006-8993(99)01224-x 10216183

[226] Celada P, Puig MV, Díaz-Mataix L, Artigas F. The hallucinogen DOI reduces low-frequency oscillations in rat prefrontal cortex: reversal by antipsychotic drugs. Biol Psychiatry. 2008;64:392–400. https://doi.org/10.1016/j.biopsych.2008.03.013 18436196

[227] Puig MV, Celada P, Díaz-Mataix L, Artigas F. In vivo modulation of the activity of pyramidal neurons in the rat medial prefrontal cortex by 5-ht2a receptors: relationship to thalamocortical afferents. Cerebral Cortex (New York, N.Y. : 1991). 2003;13:870–882. https://doi.org/10.1093/cercor/13.8.870 12853374

[228] Riga MS, Soria G, Tudela R, Artigas F, Celada P. The natural hallucinogen 5-MeO-DMT, component of Ayahuasca, disrupts cortical function in rats: reversal by antipsychotic drugs. Int J Neuropsychopharmacol. 2014;17:1269–1282. https://doi.org/10.1017/S1461145714000261 24650558

[229] Araneda R, Andrade R. 5-Hydroxytryptamine2 and 5-hydroxytryptamine 1A receptors mediate opposing responses on membrane excitability in rat association cortex. Neuroscience. 1991;40:399–412. https://doi.org/10.1016/0306-4522(91)90128-b 1851255

[230] Amargós-Bosch M, Bortolozzi A, Puig MV, et al Co-expression and in vivo interaction of serotonin1A and serotonin2A receptors in pyramidal neurons of prefrontal cortex. Cereb Cortex. 2004;14:281–299. https://doi.org/10.1093/cercor/bhg128 14754868

[231] Winters WD. The continuum of CNS excitatory states and hallucinosis. In: Hallucinations. John Wiley and Sons; 1975:53–70.

[232] Jardri R, Hugdahl K, Hughes M, et al Are hallucinations due to an imbalance between excitatory and inhibitory influences on the brain? Schizophr Bull. 2016;42:1124–1134. https://doi.org/10.1093/schbul/sbw075 27261492 PMC4988749

[233] Savalia NK, Shao LX, Kwan AC. A dendrite-focused framework for understanding the actions of ketamine and psychedelics. Trends Neurosci. 2021;44:260–275. https://doi.org/10.1016/j.tins.2020.11.008 33358035 PMC7990695

[234] Shukla R, Watakabe A, Yamamori T. mRNA expression profile of serotonin receptor subtypes and distribution of serotonergic terminations in marmoset brain. Front Neural Circuits. 2014;8:52. https://doi.org/10.3389/fncir.2014.00052 24904298 PMC4032978

[235] Watakabe A, Komatsu Y, Sadakane O, et al Enriched expression of serotonin 1B and 2A receptor genes in macaque visual cortex and their bidirectional modulatory effects on neuronal responses. Cerebral Cortex (New York, N.Y. : 1991). 2009;19:1915–1928. https://doi.org/10.1093/cercor/bhn219 19056862 PMC2705701

[236] Aqil M, Roseman L. More than meets the eye: the role of sensory dimensions in psychedelic brain dynamics, experience, and therapeutics. Neuropharmacology. 2023;223:109300. https://doi.org/10.1016/j.neuropharm.2022.109300 36334767

[237] Zarkali A, Luppi AI, Stamatakis EA, et al Changes in dynamic transitions between integrated and segregated states underlie visual hallucinations in Parkinson’s disease. Commun Biol. 2022;5:1–15. https://doi.org/10.1038/s42003-022-03903-x 36075964 PMC9458713

